# Cationic nanoparticles with disrupting neutrophil extracellular traps inhibit the progression of head and neck squamous cell carcinoma

**DOI:** 10.3389/fcell.2026.1803439

**Published:** 2026-04-22

**Authors:** Zhaoqiang Zhang, Yujie Kang, Bingxu Lu, Guichao Zhang, Baohan Xie, Yunyi Wang

**Affiliations:** 1 School of Materials Science and Engineering, Key Laboratory for Polymeric Composite and Functional Materials of Ministry of Education, Sun Yat-sen University, Guangzhou, China; 2 Department of Oral and Maxillofacial Surgery, Stomatological Hospital, School of Stomatology, Southern Medical University, Guangzhou, China

**Keywords:** cationic nanoparticles, head and neck squamous cell carcinoma, metastasis, neutrophil extracellular traps, tumor microenvironment

## Abstract

**Introduction:**

Immune checkpoint inhibitors (ICIs) have demonstrated promising therapeutic potential in head and neck squamous cell carcinoma (HNSCC). However, their clinical efficacy remains limited due to low response rates, largely attributed to the highly immunosuppressive tumor microenvironment (TME). Neutrophil extracellular traps (NETs) have recently been implicated in tumor progression and immune regulation. Therefore, this study aims to investigate the role of NETs in the TME and metastasis of HNSCC, and then to utilize cationic nanoparticles (cNP) to efficiently disrupt NETs in order to improve the immunosuppressive TME and inhibit metastasis, thereby suppressing the progression of HNSCC.

**Methods:**

Histological and bioinformatics analyses were performed to evaluate NET-related signatures in HNSCC tissues and their association with immune cell infiltration.* In vitro* (scratch, cell adhesion and cytoskeletal remodeling assay) and *in vivo* (flow cytometry, immunofluorescence staining) experiments were conducted to investigate the effects of cationic nanoparticles (cNP) on NETs disruption, tumor cell migration, therapeutic efficacy and modulation of the TME. The underlying mechanism by which NET-DNA promotes HNSCC cell migration via CCDC25 was assessed using multiple approaches. Specifically, histological and immunohistochemical staining, western blotting, transfection, qRT-PCR, and transwell assays were performed.

**Results:**

Histological analysis revealed that the expression level of NETs in HNSCC tissue was significantly higher than that in adjacent normal tissue. Bioinformatics analysis revealed that high expression of myeloperoxidase (MPO) gene is closely related to immunosuppressive cell infiltration. Further analysis revealed that arm-level gain of the MPO gene was significantly correlated with a decrease in the infiltration levels of various immune cells. *In vitro* and *in vivo* experiments demonstrated that cNP effectively disrupted NETs, inhibited tumor cell migration, and reversed the immunosuppressive TME. Compared with anti-PD-1 monotherapy, cNP combined with anti-PD-1 therapy synergistically inhibited tumor growth, prolonged survival, and significantly increased the proportion of CD8^+^ T cells and CD19^+^ B cells, while simultaneously inhibiting the infiltration levels of Treg cells and M-MDSCs, and promoting macrophage polarization from M2 to M1 types, significantly alleviating the immunosuppressive state of TME. Immunofluorescence staining further confirmed that cNP or cNP combined with anti-PD-1 therapy effectively reduced NET-DNA deposition in tumor tissue. Regarding migration mechanisms, NET-DNA can promote HNSCC cell migration through its receptor CCDC25.

**Discussion:**

This study reveals the key role of NETs in immune escape and metastasis of HNSCC and confirms that cNP disrupting NETs can synergistically enhance the efficacy of anti-PD-1 therapy, providing a new combination therapy strategy for HNSCC.

## 1 Introduction

Head and neck squamous cell carcinoma (HNSCC) is the most common malignant tumor of the head and neck region and primarily originates from the mucosal epithelium of the oral cavity and pharynx, posing a serious threat to global health. According to statistics, approximately 900,000 new cases of HNSCC and about 500,000 related deaths were reported worldwide in 2020, with a marked male predominance, making HNSCC the eighth most common cancer among men in the United States (Bhatia and Burtness, 2023). Data from the American Cancer Society and the National Cancer Registry indicate that by 2025, approximately 60,000 new HNSCC cases and nearly 12,000 deaths are expected annually in the United States (Siegel et al., 2025). HNSCC is characterized by high aggressiveness, with rapid local invasion, frequent lymph node metastasis (Jimenez et al., 2015; Wu et al., 2021), and a profoundly immunosuppressive tumor microenvironment (TME) (Denaro et al., 2021; Hong et al., 2024), all of which substantially increase therapeutic difficulty and the risk of poor prognosis.

HNSCC is widely regarded as a malignancy with impaired immune surveillance, and therefore immune checkpoint inhibitors (ICIs) have shown considerable therapeutic potential (Kitamura et al., 2020). Among these, the programmed death ligand-1 (PD-L1)/programmed death-1 (PD-1) signaling pathway plays a pivotal role in tumor immune evasion. The interaction between PD-1 and PD-L1 suppresses T-cell–mediated immune responses and represents a key mechanism by which tumor cells escape immune surveillance. Given that HNSCC tumor cells often exhibit high PD-L1 expression, anti-PD-L1 antibodies have demonstrated notable therapeutic advantages in HNSCC treatment (Cohen et al., 2019). However, the overall clinical response rate to PD-1/PD-L1–based immunotherapy remains limited. Previous studies have shown that only approximately 15% of patients with HNSCC respond to PD-1/PD-L1 inhibitors. Moreover, current research on immunotherapy resistance has largely focused on alterations in T cells and B cells, while relatively little attention has been paid to tumor cell–intrinsic immune resistance mechanisms and the potential impact of the tumor microenvironment on anti-PD-L1 therapeutic efficacy (Miraki Feriz et al., 2023). Various components of the TME, including immune cells, exosomes, and cytokines, can regulate PD-L1 expression and thereby influence tumor immune evasion. Emerging evidence suggests that inflammatory factors within the TME may represent key determinants of the therapeutic response to PD-1/PD-L1 blockade (Jiang et al., 2019). Therefore, in-depth investigation of the TME may contribute to improving the efficacy of PD-1/PD-L1–based immunotherapy.

Neutrophil extracellular traps (NETs) are web-like structures released by neutrophils in response to infection or other stimuli and are primarily composed of deoxyribonucleic acid (DNA), histones, granular proteins, and enzymes. The primary function of NETs is to capture and neutralize invasive pathogens. Although NETs exert antimicrobial effects during infection, excessive NETs formation can trigger pro-inflammatory responses, leading to host tissue damage (Liang et al., 2023). NETs contribute to the establishment of immunosuppressive conditions within the TME through multiple mechanisms in the following aspects, thereby enhancing tumor immune evasion. (1) NETs-induced CD8^+^ T-cell exhaustion: CD8^+^ T cells are among the most effective effector cells in antitumor immunity and have a significant impact on prognosis in various cancers (Raskov et al., 2021; Berger-Achituv et al., 2013). Studies have demonstrated that NETs may directly induce T-cell exhaustion via the PD-1/PD-L1 axis. Accordingly, targeting PD-L1 within NETs has been shown to restore T-cell function and alleviate tumor burden (Kaltenme et al., 2021). Mechanistically, NET-derived DNA can bind to transmembrane and coiled-coil domains 6 (TMCO6) on CD8^+^ T cells, thereby impairing antitumor immunity and promoting hepatocellular carcinoma (HCC) progression (Song et al., 2024). (2) Physical barrier formation: NETs can form a physical barrier at the tumor–stroma interface, protecting tumor cells from CD8^+^ T-cell infiltration into tumor regions and consequently weakening T-cell cytotoxic activity (Fang et al., 2022). (3)Regulation of Treg differentiation: Regulatory T cells (Tregs) are an immunosuppressive subset of CD4^+^ T cells and play a critical role in the tumor immune microenvironment. NETs can induce Treg differentiation from naïve CD4^+^ T cells by enhancing the oxidation of NADH to NAD^+^ and reprogramming oxidative phosphorylation (OXPHOS), which is essential for Treg differentiation (Fang et al., 2022; Wang et al., 2021). Moreover, neutrophils can recruit macrophages and Treg cells to promote the growth of HCC (Zhou et al., 2016). In HNSCC, high levels of Treg infiltration have been detected (Ou et al., 2017), and elevated Treg levels in the peripheral blood of HNSCC patients are associated with immunosuppression and poor prognosis (Jie et al., 2013). It has been demonstrated that modulation of Treg function can suppress HNSCC progression (Rutihinda et al., 2023). (4) Effects on B cells: CD19 is a transmembrane protein expressed on the surface of B cells and is closely associated with B-cell proliferation, activation, signal transduction, and growth regulation. NET-derived DNA influences B-cell activation and tolerance mechanisms, in which the methylation status of CpG motifs and their interaction with Toll-like receptors (TLRs) play critical roles (Wang and Krieg, 2003). Furthermore, in combined treatment with PD-1 inhibitors in HNSCC, increased numbers of B cells and effector T cells have been observed in the TME (Luginbuhl et al., 2022). (5) Macrophage polarization: Macrophages represent the most abundant innate immune cell population within the tumor immune microenvironment (TIME). They can be broadly classified into antitumor M1 macrophages and protumor M2 macrophages. During tumor initiation and progression, peripheral blood monocytes are recruited to tumor tissues under the influence of various chemokines and differentiate into tumor-associated macrophages (TAMs) (Wei et al., 2019). Accumulating evidence indicates that TAMs promote HNSCC growth, invasion, and metastasis (She et al., 2018; Gao et al., 2018). NETs can drive macrophage polarization and frequently rely on macrophages to facilitate cancer cell invasion and metastasis (Fang et al., 2022). In an *in vitro* co-culture system of macrophages and A549 lung cancer cells, the addition of purified NETs significantly enhanced cancer cell migration and invasion (Zhang et al., 2022). Moreover, studies on the interaction between macrophages and neutrophils undergoing NETosis have shown that, following NET degradation, macrophages exhibit phenotype-dependent responses: after several hours of interaction, M2 macrophages induce pro-inflammatory responses, whereas M1 macrophages undergo cell death accompanied by nuclear decondensation (Nakazawa et al., 2016). (6) Induction of myeloid-derived suppressor cells (MDSCs): Within the TME, malignant cells primarily evade immune surveillance by increasing the generation of pathologically activated MDSCs. It has been reported that cytotoxic T and B cells are associated with prolonged survival, whereas elevated densities of Treg cells and MDSCs correlate with poor prognosis (Raskov et al., 2022). MDSCs are precursor cells of macrophages, dendritic cells, and granulocytes and can be classified into polymorphonuclear MDSCs (PMN-MDSCs, also referred to as G-MDSCs) and monocytic MDSCs (M-MDSCs) based on surface marker expression patterns (Veglia et al., 2021). In HNSCC, MDSCs exhibit strong immunosuppressive properties, and their accumulation is positively correlated with tumor progression, suppression of cytotoxic responses, increased metastatic potential, and unfavorable clinical outcomes (Keskinov and Shurin, 2015; Bronte et al., 2016).MDSCs contribute to tumor progression by non-specifically suppressing T-cell responses (Riquelme, 2020). They can induce tolerance to multiple antitumor effector cells, including CD4^+^ and CD8^+^ T cells (Solito et al., 2012). Additionally, MDSCs inhibit T-cell proliferation through multiple mechanisms, such as the expression of arginase-1 (ARG1) and inducible nitric oxide synthase (iNOS), depletion of L-arginine, and production of nitric oxide (NO), thereby suppressing T-cell function (Jayaraman et al., 2018).

In addition to their close association with tumor immune evasion, NETs are also critically involved in cancer metastasis. Numerous studies have demonstrated that NET-derived DNA serves as a ligand for the cancer cell transmembrane protein CCDC25 and can specifically bind to it. Following NET-DNA–CCDC25 interaction, intracellular signaling pathways are activated, particularly the ILK–β-parvin pathway, leading to the activation of small GTPases such as RAC1 and CDC42. This cascade promotes cytoskeletal remodeling and ultimately enhances cancer cell migration and motility (Yang et al., 2020) (Liu et al., 2020; Milette et al., 2020). Conversely, metastatic cancer cells can induce neutrophils to release NETs, thereby further promoting tumor metastasis; these processes mutually reinforce each other during cancer progression (Liang et al., 2023; Yang et al., 2020; Park et al., 2016).

In summary, NETs promote immune evasion of HNSCC cells, form physical barriers that impede immune cell infiltration, and enhance tumor cell motility and metastasis. Therefore, clearance of NETs represents a promising strategy to improve the efficacy of immunotherapy in HNSCC. It has been demonstrated that cNP can effectively eliminate circulating free DNA (cfDNA). Our group previously prepared a series of cNP uniformly coated with single or di-hydroxyl groups and different types of amino groups, and the results showed that hydroxylated nanoparticles exhibited prolonged *in vivo* retention and enhanced cfDNA clearance capacity, ultimately improving therapeutic outcomes (Liu et al., 2022). Accordingly, the present study aims to alleviate the immunosuppressive TME in HNSCC by eliminating NETs and disrupting physical barriers, thereby inhibiting immune evasion by HNSCC cells. Moreover, removal of NETs can block the interaction between NET-DNA and CCDC25, suppressing NET-DNA–mediated cancer cell migration and metastasis in HNSCC and ultimately restraining disease progression while enhancing immunotherapeutic efficacy. On this basis, we further combined cNP with anti-PD-1 antibodies to improve the therapeutic performance of PD-1/PD-L1 blockade in HNSCC. Collectively, this study not only elucidates the interplay between NETs, immune dysregulation, and metastasis in HNSCC but also proposes an integrated “NETs–immune checkpoint–metastasis” therapeutic targeting strategy, providing new insights for the clinical treatment of HNSCC.

## 2 Materials and methods

### 2.1 Materials and reagents

Fetal Bovine Serum (FBS), Dulbecco’s Modified Eagle Medium/Nutrient Mixture F-12 (DMEM/F-12) medium, Dulbecco’s Modified Eagle Medium (DMEM), Roswell Park Memorial Institute 1640 Medium (RPMI 1640), 4% paraformaldehyde (biosharp), Phosphate buffered solution and Opti-MEM were purchased from Gibco. Alexa Fluor™ 750-NHS, TRIzol and Quant-iT PicoGreen dsDNA Assay Kit were purchased from Invitrogen. MicroElute DNA Clean Up Kit was purchased from Omega. Normocin and phorbol myristate acetate (PMA) was supplied by InvivoGen. Bovine serum albumin (BSA) was purchased Sigma-Aldrich. All-in-One First-Strand Synthesis kit (Catalog No. GXRT003, GenXion Biotechnology, China), 2X GenXion SYBR Green qPCR Premix (Catalog No. JXP9009, GenXion Biotechnology, China), Cell Counting Kit-8 (GenXion Biotechnology, China), Anti-PD-1 antibody (Catalog No. BE0273, BioXcells), Human Peripheral Blood Neutrophil Isolation Kit (Catalog No. LZS11131, Haoyang), CCDC25 antibody (Catalog No. 21209-1AP, Proteintech), Anti-Histone H3 (citrulline R2 + R8 + R17) (H3cit) antibody (Catalog No. ab5103, Abcam), human/mouse myeloperoxidase/MPO antibody (Catalog No. AF3667, R&D), donkey anti-goat IgG H&L (Alexa Fluor 647) (Catalog No. ab15031, Abcam), goat anti-rabbit IgG H&L (Alexa Fluor 488) (Catalog No. ab150077), Alexa Fluor 555 Phalloidin (Thermo Fisher), Universal two-step test kit (Mouse/rabbit enhanced polymer detection system) (Catalog No. PV-9000, ZSGB-BIO), Lipofectamine 2000 (Thermo Fisher), Penicillin-Streptomycin (Thermo Fisher), HRP-conjugated monoclonal mouse anti-GAPDH (Catalog No. KC-5G5; KangCheng Bio-tech, China), HRP Conjugated AffiniPure Goat Anti-rabbit IgG (H + L) (Catalog No. BA1054; Boster Biological Technology, China), Zombie Aqua Fixable Viability Kit (Catalog No. 423102, BioLegend), APC anti-mouse CD19 Antibody (Catalog No. 115512, BioLegend), Brilliant Violet 421 anti-mouse CD45 (Catalog No. 103134, BioLegend), Alexa Fluor 700 anti-mouse CD3 (Catalog No. 100216, BioLegend), PE/Cyanine7 anti-mouse CD4 (Catalog No. 100422, BioLegend), APC anti-mouse CD25 Antibody (Catalog No. 102011, BioLegend), PE anti-mouse CD8a Antibody (Catalog No. 100708, BioLegend), FITC anti-mouse F4/80 Antibody (Catalog No. 123107, BioLegend), Brilliant Violet 711 anti-mouse/human CD11b Antibody (Catalog No. 101242, BioLegend), APC anti-mouse CD86 Antibody (Catalog No. 105012, BioLegend), CD163- APC/Cy7 (Catalog No. 155324, BioLegend), PE/Dazzle 594 anti-mouse Ly-6C Antibody (Catalog No. 128043, BioLegend), Alexa Fluor 700 anti-mouse Ly-6G (Catalog No. 127622, BioLegend), Purified anti-mouse CD16/32 (Catalog No. 101302, BioLegend), FOXP3 Monoclonal Antibody (FJK-16s), FITC (Catalog No. 11-5773-82, eBioscience) were purchased.

### 2.2 Preparation and testing the properties of cNP

**FIGURE 1:**
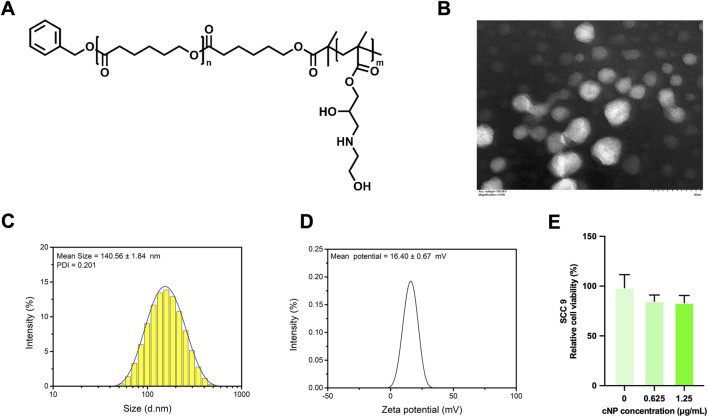
**(A)** Structural formula of PCL60-b-PGEA150. **(B)** Transmission electron microscopy (TEM) image of cNP (scale bar: 50 nm). **(C)** Particle size distribution curve of cNP in PBS determined by dynamic light scattering (DLS) (n = 3). **(D)** Zeta potential of cNP in PBS (n = 3). **(E)** Cell viability of SCC9 cells treated with different concentrations of cNP, assessed by the CCK-8 assay (n = 3, means ± s.d.).

The cNP used in this study were kindly provided by members of our research group. Their preparation procedure was conducted according to our previously published work (Liu et al., 2022). Briefly, PCL_60_-*b*-PGEA_150_ was synthesized (Figure 1A) and subsequently self-assembled into cNP. Specifically, PCL_60_-*b*-PGEA_150_ (20 mg) was dissolved in 1 mL of N,N-dimethylformamide (DMF). Under ultrasonication (Sonics VCX105, 20 kHz, 70% power level) in an ice bath, the DMF solution was added dropwise into 9 mL of phosphate-buffered saline (PBS). The resulting solution was dialyzed against PBS (pH = 7.4) to remove DMF. The obtained cNP were characterized by transmission electron microscopy (TEM), nanoparticle size analysis, and zeta potential measurement.

### 2.3 Cell culture

Human tongue squamous-cell carcinoma cell lines used in this study included SCC9, SCC15, Cal27 and Cal33. Furthermore, the SCCVII is a murine squamous cell carcinoma line and derived from C3H. SCC 9, SCC15, Cal27 were a kind gift from The First Affiliated Hospital, Sun Yat-sen University. Cal33 and SCCVII was a kind gift from Hospital of Stomatology, SunYat-sen University. Human oral keratinocytes (HOK) was purchased from Tong Pai Technology (Shanghai, China). The SCC9, SCC15, Cal27 and Cal33 cells were maintained in culture medium, which consist of DMEM, 10% fetal bovine serum (FBS) and Normocin, and cultured in a humidified condition including 5% CO_2_ at 37 °C. The SCCVII cells is cultured in DMEM/F-12 medium containing 10% FBS and 0.4% penicillin-streptomycin solution. HOK cells were cultured in DMEM high-glucose medium containing 10% FBS and 1% penicillin/streptomycin solution.

### 2.4 Isolation and purification of NET-DNA

The isolation and purification of NETs were performed as previously described (Warnatsch et al., 2015; Noubouossie et al., 2017) and were used for subsequent experiments involving oral cancer cells. NETs were isolated from primary human neutrophils obtained from the peripheral blood of healthy donors. This study was approved by the Medical Ethics Committee of the Stomatological Hospital of Southern Medical University (Approval No. NYKQ-EC-[2024] 45). Briefly, human neutrophils were isolated using a Human Peripheral Blood Neutrophil Isolation Kit. The collected neutrophils were cultured in RPMI 1640 medium supplemented with 1% bovine serum albumin (BSA) and 500 nM phorbol 12-myristate 13-acetate (PMA) for 4 h in a humidified incubator at 37 °C with 5% CO_2_. After 4 h, NETs were formed and adhered to the bottom of the culture plates. The remaining culture medium was carefully removed, and the NETs were gently washed with 2 mL of cold phosphate-buffered saline (PBS). The suspension was then centrifuged at 1,000 × g for 10 min at 4 °C, and the cell-free supernatant containing NETs was collected. According to a previously reported protocol (Yang et al., 2020), NETs were further fragmented using an ultrasonic processor (Sonics VCX105). NET-DNA was subsequently purified using a MicroElute DNA Clean-Up Kit. Finally, the concentration of NET-DNA was quantified using a Quant-iT PicoGreen dsDNA Assay Kit. The NET-containing supernatant was stored at −20 °C until further use.

### 2.5 Cell counting kit (CCK)-8 assay

Cell viability was evaluated using the CCK-8 assay. SCC9, SCC15, Cal27, and Cal33 cells (1 × 10^4^ cells/mL) were seeded into 96-well plates and incubated at 37 °C with 5% CO_2_ for 12 h. According to the experimental grouping, cells were treated with different concentrations of NET-DNA or cNP. After incubation for 48 h, CCK-8 reagent was added to each well and incubated at 37 °C for 1 h. The absorbance at 450 nm (A_450_) was measured using a microplate reader (Rayto RT-6000). Cell viability was calculated using Equation 1, where A_t_ represents the A_450_ value of cancer cells treated with NET-DNA or cNP, A_c_ represents the A_450_ value of cancer cells treated with culture medium alone (blank group), and A_b_ represents the A_450_ value of the blank control.

$$\text{Cell}\;\text{viability}\left(\%\right)=\frac{A_{t}-A_{b}}{A_{c}-A_{b}}\times 100\%$$ (1)

### 2.6 Wound healing assay *in vitro*

The wound healing assay was performed to evaluate SCC9 cell migration under stimulation with different concentrations of NET-DNA or cNP. When SCC9 cells reached approximately 80% confluence, a linear scratch was generated using a 200 μL pipette tip, and detached cells were removed by washing with PBS. Cells were then incubated with medium containing different concentrations of NET-DNA or cNP at 37 °C. Images were captured at 0, 24, and 48 h using an inverted optical microscope. The wound healing area was quantified using ImageJ software according to Equation 2.

$$\text{Healing area }\left(\%\right)=\frac{\text{Healing}\;\text{area}\;\text{on}\;\text{the}\;\text{indicated}\;\text{day}}{\text{Original}\;\text{wound}\;\text{area}}\times 100\%$$ (2)

### 2.7 Cell adhesion and cytoskeletal remodeling assay

Cell adhesion and cytoskeletal remodeling assays were conducted to evaluate the adhesion capacity and cytoskeletal changes of SCC9 cells following stimulation with NET-DNA (1 μg/mL) and different concentrations of cNP. NET-DNA or cNP (100 μL per well) were added to 96-well plates and incubated overnight at 37 °C. After removal of the supernatant, 100 μL of serum-free medium containing 5 × 10^4^ cells was added to each well and incubated at 37 °C for 15 min. Cells were fixed with 4% paraformaldehyde at room temperature for 20 min and stained with crystal violet at 4 °C overnight. Adherent cells were observed and imaged using phase-contrast microscopy (Leica). Three fields per well were randomly selected, and adherent cells were counted using ImageJ software.

For cytoskeletal remodeling analysis, control and treated SCC9 cells (2 × 10^3^ cells/well) were evenly seeded in 48-well plates and cultured overnight. After removal of the culture medium, cells were incubated with serum-free medium containing NET-DNA and different concentrations of cNP for 3 h. Cells were fixed with 4% paraformaldehyde for 15 min, permeabilized with 0.1% Triton X-100 for 15 min, and stained with DAPI for nuclear visualization. Filamentous actin (F-actin) was stained with Alexa Fluor 555–conjugated phalloidin. Cells were observed using a confocal laser scanning microscope (Leica SP8). For quantification, filopodium-like protrusions (FLPs) were counted from 10 fields per well, and three independent biological experiments were performed.

### 2.8 Histological and immunohistochemical staining

This parts aimed to determine the expression level of CCDC25 protein in HNSCC tissues and adjacent non-tumor tissues. This study was approved by the Medical Ethics Committee of the Stomatological Hospital of Southern Medical University (Approval No. NYKQ-EC-[2024] 45). After surgical resection, tumor tissues from HNSCC patients were fixed in neutral buffered formalin, dehydrated using an automated vacuum tissue processor (Leica ASP200S), embedded in paraffin using an embedding system (Leica, EG1150), and sectioned into 4 μm-thick slices using a microtome (Leica RM2245). For histological analysis, deparaffinized and rehydrated sections were stained with hematoxylin and eosin (H&E). For immunohistochemical (IHC) staining, paraffin-embedded sections (4 μm) were deparaffinized in xylene and rehydrated through graded ethanol. Antigen retrieval was performed in 0.01 M sodium citrate buffer (pH 6.0) by microwave heating for 15 min, followed by natural cooling to room temperature. Endogenous peroxidase activity was blocked with 3% H_2_O_2_ for 20 min at room temperature. After washing with PBS, sections were blocked with 10% normal goat serum for 30 min. Sections were then incubated with a primary antibody against CCDC25 (1:50) at 4 °C overnight. After PBS washing, sections were incubated with a mouse anti-rabbit IgG secondary antibody (universal two-step detection kit) at 37 °C for 30 min. Signal development was performed using DAB substrate, followed by hematoxylin counterstaining. Sections were dehydrated, cleared, mounted, and observed under a light microscope. Five randomly selected high-power fields were analyzed, and positive staining was semi-quantitatively evaluated using ImageJ software.

### 2.9 Immunofluorescence staining

For NET-DNA immunofluorescence staining, tissue sections were deparaffinized, rehydrated, and permeabilized with 0.025% Triton X-100 for 5 min. Non-specific binding sites were blocked with 1% BSA. Sections were then incubated with primary antibodies against H3cit (1:100) and MPO (1:100) at 4 °C for 24 h, followed by incubation with Alexa Fluor 488– and Alexa Fluor 647–conjugated secondary antibodies (1:1000) at 37 °C for 1.5 h. Nuclei were counterstained with DAPI. Slides were scanned and imaged using an inverted confocal microscope (Leica). NET formation was quantified as the percentage of H3cit-positive area relative to the total tissue area using ImageJ software, according to a previously described method (Yang et al., 2020). For CCDC25 immunofluorescence staining, cells were seeded on coverslips and fixed with 4% paraformaldehyde for 20 min. Cells were permeabilized with 0.1% Triton X-100 for 20 min and blocked with 5% BSA for 30 min at room temperature. Cells were incubated with a primary antibody against CCDC25 (1:100) at 4 °C overnight, followed by incubation with Alexa Fluor 488–conjugated secondary antibody (1:1000) at 37 °C for 1 h. Nuclei were stained with DAPI, and images were acquired using a confocal microscope.

### 2.10 Western blot analysis

Western blot analysis was performed to evaluate CCDC25 expression in different cell lines. Proteins were extracted from cultured SCC9, SCC15, Cal27, and Cal33 cells using RIPA lysis buffer and incubated on ice for 30 min. Protein concentrations were determined using a BCA protein assay kit. Equal amounts of total protein (50 μg per lane) were separated by SDS–PAGE and transferred onto PVDF membranes. Membranes were blocked with 5% BSA in TBST at room temperature and incubated with primary antibodies against GAPDH (1:10,000) and CCDC25 (1:500) at 4 °C overnight. After washing with TBST, membranes were incubated with secondary antibodies (1:10,000) for 1 h. Protein bands were visualized using enhanced chemiluminescence (ECL) reagents and imaged with a KwikQuant digital imaging system. Band intensity was quantified using SensiAnsys software on a JS-680A automatic gel imaging system.

### 2.11 Transfection and qRT-PCR analysis

SCC9 cells (0.5 × 10^5^) were seeded in 400 μL antibiotic-free medium and transfected with siRNA (sequences listed in Supplementary Table S1) when cell confluence reached 30%–50%. siRNA and Lipofectamine 2000 were diluted in Opti-MEM, mixed, and added to 24-well plates. Cells were incubated at 37 °C with 5% CO_2_ for 24 h. After transfection, CCDC25 mRNA expression was analyzed by qRT-PCR. Total RNA was isolated using TRIzol reagent, and cDNA was synthesized using an All-in-One First-Strand Synthesis Kit. Quantitative PCR was performed using 2× GenXion SYBR Green qPCR Premix on a QuantStudio 1 Real-Time PCR System, with GAPDH as an internal control. Primer sequences are listed in Supplementary Table S2. All samples were analyzed in triplicate.

### 2.12 Transwell assay

At 48 h post-transfection, SCC9 cells were collected for transwell migration assays to assess the effect of CCDC25 knockdown on NET-DNA–induced cell migration. Cells (5 × 10^4^) were resuspended in serum-free DMEM and seeded into the upper chambers of transwell inserts with an 8 μm pore size. The lower chambers were filled with 500 μL serum-free medium with or without 1 μg/mL NET-DNA. After 24 h of incubation, migrated cells on the lower surface of the membrane were fixed with 4% paraformaldehyde and stained with 0.2% crystal violet. Migrated cells were counted in five randomly selected fields under a microscope.

### 2.13 Animal experiments

Animal experiments were approved by the Institutional Animal Care and Use Committee of Sun Yat-sen University (Approval No. SYSU-IACUC-2025-000382). Female C3H mice (5–8 weeks) were purchased from Shanghai Model Organisms Center, Inc. All mice were bred and used in the specific-pathogen free (SPF) animal facility of the Animal Experiment Center of Sun-Yat-Sen University. The cells (5 × 10^5^ cells in 100 μL FBS- free DMEM/F-12 medium) were injected subcutaneously into the shaved right flanks of C3H mice. The day of inoculation was day 0. Thereafter, all the treated C3H mice were monitored every day and measured the tumor sizes [Tumor volume (mm^3^) = 0.5 × length × width^2^] by using the vernier caliper every 2 days. When the tumor volume of the mice reached to 50–80 mm^3^ on day 7, the administration models were treated with PBS, cNP (15 mg/kg, i.v., refer to previous research (Liu et al., 2022)), anti-PD-1 (5 mg/kg, i.p.), cNP plus anti-PD-1 on day 7, 9, 12, and 15 (Figure 7A). On the day 22, five mice from each group were executed and tumor tissues were taken for immuno-microenvironmental analysis and immunofluorescence staining for observe the expression of NET-DNA in cancer tissues. Seven mice in each group were used individually for the survival analysis by using Kaplan-Meier method.

### 2.14 Immuno-microenvironmental analysis *in vivo* via flow cytometry

On the day 22, five mice from each group were sacrificed and their tumor tissues were evaluated for immune cell infiltration. Tumor tissues were cut into small pieces (2–4 mm), then ground and passed through a sieve screen to obtain a tumor single cell suspension. Cells were stained for viability by using the Zombie Aqua Fixable Viability Kit in order to distinguish live/dead populations. Then the non-specific binding of antibodies to Fc receptors was blocked with anti-mouse CD16/32 antibodies at 4 °C for 30 min. Subsequently, the cells were incubated with the surface marker staining fluorescent antibodies including CD45, CD3,CD4,CD8a,CD25,CD19,F4/80,CD11b,CD86,CD163,LY-6C,LY-6G. To wash and resuspend the cells after the staining. The cells were fixed with 500 μL fixation buffer and then permeabilized. The intracellular staining was performed with FOXP3 antibody for 30 min at 4 °C after washing twice with permeabilization wash buffer. Finally, Cells were washed twice with permeabilization wash buffer after staining and then resuspended with FACS buffer. All samples were analyzed by using the Attune NxT Acoustic Focusing Cytometer (Thermo Fisher Scientific, United States) and the data quantitative analysis was performed with FlowJo software.

### 2.15 Bioinformatics analysis

#### 2.15.1 Data acquisition

Transcriptomic and copy number variation (CNV) data were obtained from the UCSC Xena platform (https://xenabrowser.net). RNA-seq data for the TCGA Head and Neck Squamous Cell Carcinoma (TCGA-HNSC) cohort were downloaded from the TCGA Hub (dataset: “TCGA.HNSC.sampleMap/HiSeqV2”, IlluminaHiSeq_RNASeqV2, n = 566). The data represent Level 3 RSEM-normalized counts and were log_2_ (x + 1) transformed prior to analysis. Gene annotations were mapped using the UCSC Xena HUGO probeMap (hg19). Gene-level CNV data were retrieved from the TCGA-HNSC GISTIC2-processed dataset (“TCGA.HNSC.sampleMap/Gistic2_CopyNumber_Gistic2_all_data_by_genes”, n = 522). Thresholded CNV values were classified as Deep Deletion, Arm-level Deletion, Diploid/Normal, or Arm-level Gain. Single-cell RNA-seq data were obtained from the GEO database (GSE181919) for validation analyses (Choi et al., 2023). All datasets were matched using sample barcodes, and samples with missing key variables were excluded prior to downstream analyses.

#### 2.15.2 Immune infiltration analysis

To assess the composition of immune cells within the tumor microenvironment, the CIBERSORT algorithm was employed to perform deconvolution analysis on tumor samples (Newman et al., 2015). The normalized TPM expression matrix was used as input, together with the LM22 signature gene matrix provided by the developers of CIBERSORT, to estimate the relative proportions of 22 human immune cell types. The analysis was conducted in R using the official CIBERSORT script, with 1,000 permutations (permutations = 1000) and quantile normalization enabled (quantile normalization = TRUE) to enhance comparability across samples.

#### 2.15.3 Correlation analysis

Pearson correlation analysis was performed using the cor and cor.test functions in R (version 4.4.0) to evaluate the strength and direction of linear relationships between immune cell proportions and gene expression variables, as well as gene–gene expression correlations. To minimize scale effects, all continuous variables were log_2_-transformed and Z-score normalized prior to correlation analysis. Only samples with complete data were included in the analysis. P-values were adjusted for multiple testing using the Benjamini–Hochberg method, and an adjusted P < 0.05 was considered statistically significant.

#### 2.15.4 Single-sample gene set enrichment analysis

To systematically evaluate the activity of key tumor-related pathways in each HNSC sample, hallmark gene sets were obtained from the Molecular Signatures Database (MSigDB v7.5.1) (Liberzon et al., 2015), including pathways such as HALLMARK_G2M_CHECKPOINT and HALLMARK_EPITHELIAL_MESENCHYMAL_TRANSITION (EMT). The GSVA R package (version 1.52.3) was used to perform single-sample gene set enrichment analysis (ssGSEA) on the normalized transcriptomic expression matrix, generating an enrichment score for each pathway within each sample.

The expression matrix was log_2_ (TPM +1) transformed prior to analysis. Default GSVA parameters were used, with the method set to “ssgsea”. The resulting enrichment scores were subsequently correlated with gene expression levels (e.g., MPO, CCDC25) to explore potential functional associations between specific genes and biological pathways.

#### 2.15.5 Immune infiltration correlation analysis

The expression level of MPO and the infiltration abundance of various immune cell subtypes were retrieved and analyzed for correlation using Pearson correlation analysis, calculating both the correlation coefficient (r) and the significance level (p). Data visualization was performed in R (version 4.4.1). In the resulting plots, bubble size represents the magnitude of correlation (|r| value), bubble color indicates the direction of correlation (positive or negative), line thickness reflects the level of statistical significance, and line color further denotes the correlation direction.

## 3 Statistical analysis

All statistical analyses were performed using R and GraphPad Prism software (version 8.0.2). Data are presented as the mean ± standard deviation (mean ± s.d.). For comparisons among multiple groups, one-way analysis of variance (ANOVA) was applied, and when significant differences were detected, Tukey’s *post hoc* test was used for pairwise comparisons. For comparisons of the same experimental subjects under different treatment conditions (e.g., time-course or intervention experiments), the paired t-test was employed. For comparisons between independent samples, the unpaired t-test was used. The P value <0.05 was considered statistically significant.

## 4 Results

### 4.1 Characterization of cNP

Transmission electron microscopy (TEM) revealed uniform spherical nanoparticles with a diameter of approximately 25 ± 5 nm, corresponding to the solid crystalline hydrophobic poly (ε-caprolactone) (PCL) core (Figure 1B). Dynamic light scattering (DLS) analysis showed a hydrated hydrodynamic diameter of approximately 140.56 nm with a unimodal size distribution (Figure 1C). This marked discrepancy between TEM and DLS measurements can be attributed to the hydrated poly (glycidyl ethylene amine) (PGEA) hydrophilic shell, which is included in DLS measurements. The abundant hydroxyl and amino groups on the PGEA chains enable extensive chain extension in aqueous environments, forming a thick solvation layer that substantially increases the hydrodynamic diameter. In addition, the zeta potential of +16.4 mV further confirmed the positively charged surface of cNP (Figure 1D), contributing to their colloidal stability. This distinct core–shell architecture and size discrepancy are characteristic features of amphiphilic block copolymer self-assemblies, indicating that the prepared cNP exhibit well-defined morphology, good dispersion stability, and favorable surface functionalization. To evaluate the cytotoxicity of cNP, a CCK-8 assay was performed to assess the effects of different concentrations of cNP (0, 0.625 and 1.25 μg/mL) on the viability of SCC9 cells (Figure 1E). The results showed a slight decrease in cell viability with increasing cNP concentration; however, no statistically significant differences were observed. These findings suggest that cNP exhibit no significant cytotoxicity toward SCC9 cells and is suitable for subsequent experiments.

### 4.2 NETs infiltration in HNSCC is more than adjacent normal tissue

**FIGURE 2:**
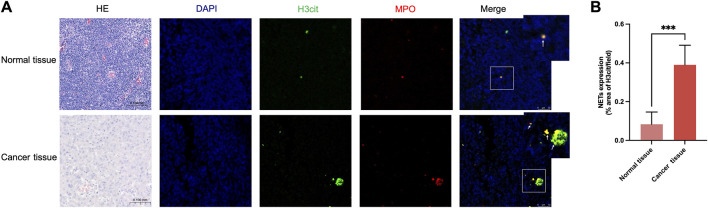
NETs infiltration in HNSCC and adjacent normal tissue. **(A)** The expression of NETs in HNSCC and adjacent normal tissue by using immunofluorescence staining for H3cit and MPO. **(B)** NETs expression quantification demonstrated that the NETs infiltration in HNSCC is more than adjacent normal tissue (n = 5, means ± s.d.; ***P < 0.001).

To investigate the expression of NETs in HNSCC, tumor tissues and matched adjacent normal tissues (n = 5) were collected. H&E staining was first performed to confirm tumor regions, followed by immunofluorescence staining (Figure 2A) using myeloperoxidase (MPO) and citrullinated histone H3 (H3Cit), which are specific markers for neutrophils and NETosis, respectively (Liang et al., 2023). NETs were scarcely detected in adjacent normal tissues, whereas a marked increase was observed in tumor tissues (Figure 2B), with statistically significant differences. These results, together with existing evidence, suggest that NETs play an important role in the TME. Accordingly, the clearance of NETs may represent a potential strategy to inhibit the progression of HNSCC.

### 4.3 NET-DNA has no significant effect on the proliferation viability of HNSCC cells

The effects of NET-DNA on cell proliferation were evaluated using a CCK-8 assay in four HNSCC cell lines (SCC9, SCC15, Cal27, and Cal33) treated with different concentrations of NET-DNA (0, 0.5, 1, and 2 μg/mL) (Supplementary Figure S1A–D). The results showed that increasing NET-DNA concentrations caused only mild changes in the proliferation of SCC9 and SCC15 cells, with no statistically significant differences. In Cal27 cells, a slight concentration-dependent promoting effect was observed, although the effect was not pronounced. In contrast, Cal33 cells exhibited a mild inhibitory trend, which was also not statistically significant. Overall, these results indicate that although different HNSCC cell lines exhibit variable responses to NET-DNA stimulation, no statistically significant differences were detected. Within the tested concentration range, NET-DNA had no significant effect on the proliferative viability of these four HNSCC cell lines, providing a basis for further investigation of the role of NET-DNA in the TME.

### 4.4 Bioinformatics methods were used to analyze the correlation between MPO and TIME of HNSCC

**FIGURE 3:**
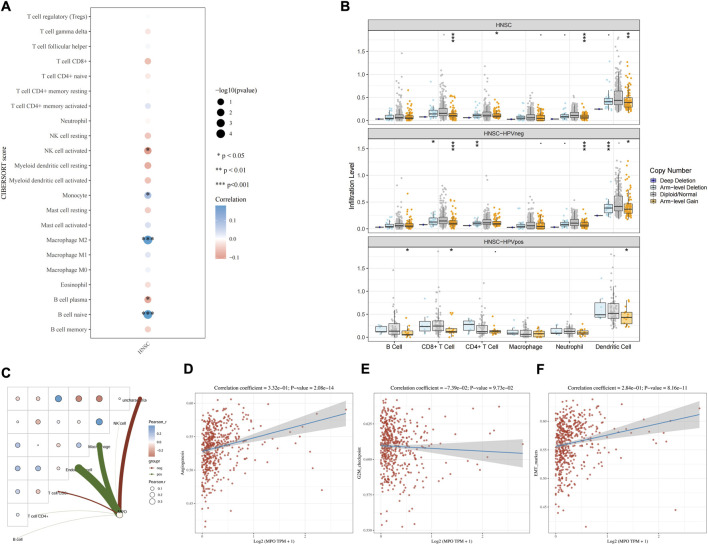
Correlation analysis of MPO and TIME of HNSCC by using bioinformatics methods. **(A)** The expression level of MPO is significantly correlated with the infiltration degree of various immune cells (*0.01 < *P* < 0.05, **0.001 < *P* < 0.01, ***P < 0.001). **(B)** The infiltration levels of immune cells in the TIME of HNSCC under different copy number variation states of MPO (*0.01 < *P* < 0.05, **0.001 < *P* < 0.01, ***P < 0.001). **(C)** Correlation analysis of MPO expression and immune cell infiltration in HNSCC. Correlation analysis of MPO expression and angiogenesis **(D)**, G2/M checkpoint **(E)** and EMT **(F)** in HNSCC.

The CIBERSORT algorithm was first applied to estimate immune cell infiltration scores in HNSCC samples. Correlation and significance analyses were performed to compare the distribution of different immune cell types. A bubble plot (Figure 3A) illustrated the correlations between MPO expression and the infiltration levels of various immune cell populations. MPO expression was positively correlated with the infiltration of monocytes, M2 macrophages, and naïve B cells, while showing negative correlations with activated NK cells and plasma B cells. These findings suggest that MPO may promote immune evasion by increasing immunosuppressive cell populations, such as M2 macrophages, while reducing immune effector cells, such as NK cells.

To further assess immune infiltration under different MPO copy number variation (CNV) states, samples were stratified into HPV-negative (HPV neg) and HPV-positive (HPV pos) subgroups. Box plots (Figure 3B) demonstrated changes in immune cell infiltration across different MPO CNV states in overall HNSCC samples and HPV-stratified subgroups. In the overall HNSCC cohort, compared with the diploid/normal group, arm-level gain of MPO was associated with significantly reduced infiltration of CD8^+^ T cells, CD4^+^ T cells, neutrophils, and dendritic cells. Similar trends were observed in the HPV-negative subgroup, whereas in the HPV-positive subgroup, arm-level gain of MPO was associated with decreased infiltration of B cells, CD8^+^ T cells, and dendritic cells. These results indicate that MPO copy number amplification may suppress the infiltration of multiple immune cell types, thereby weakening local immune responses and facilitating tumor progression. Notably, higher immune cell infiltration levels were observed in HPV-positive tumors, suggesting that HPV infection may elicit stronger immune responses. Collectively, these findings imply that MPO may exert distinct immunomodulatory effects in different HPV-related HNSCC subtypes.

Pearson correlation analysis further revealed that MPO expression was positively correlated with endothelial cells and macrophages, while negatively correlated with CD8^+^ T cells (Figure 3C). No significant correlations were observed with B cells, CD4^+^ T cells, or NK cells. These findings suggest that MPO may promote tumor progression by enhancing macrophage and endothelial cell infiltration while attenuating CD8^+^ T cell–mediated antitumor immunity.

Further pathway analyses demonstrated that MPO expression was significantly positively correlated with angiogenesis (Figure 3D) and epithelial–mesenchymal transition (EMT) (Figure 3F), whereas its correlation with the G2/M checkpoint pathway was weak and not statistically significant (Figure 3E). These results suggest that MPO may facilitate HNSCC progression primarily through regulating angiogenesis and EMT rather than cell cycle control.

### 4.5 Combination therapy with cNP and anti-PD-1 significantly inhibited the tumor growth of HNSCC-bearing mice

**FIGURE 4:**
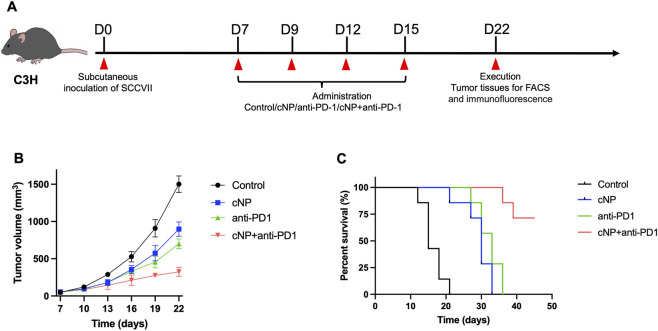
Combination therapy with cNP and anti-PD-1 significantly inhibited the tumor growth of HNSCC-bearing mice. **(A)** Experimental schedule of HNSCC-bearing mice model. **(B)** Tumor growth curves of different treatment groups (n = 5). **(C)** Survival curves of mice in different treatment groups (n = 7).

To evaluate the therapeutic efficacy of cNP in combination with anti-PD-1, a murine HNSCC model was established by subcutaneous injection of SCCVII cells into C3H mice, followed by drug administration at designated time points. The experimental design and treatment schedule are illustrated in Figure 4A, and tumor growth curves for each group are shown in Figure 4B. At the first administration time point (day 7), no significant differences in tumor volume were observed among the control, cNP, anti-PD-1, and cNP + anti-PD-1 groups. By day 10, tumor volumes in the cNP, anti-PD-1, and combination groups were significantly smaller than those in the control group, indicating that all treatments exerted antitumor effects. However, no statistically significant differences were detected among the three treatment groups. On day 13, tumor growth inhibition persisted in the cNP, anti-PD-1, and combination groups compared with the control group, while differences among the three treatment groups remained statistically insignificant. By day 16, tumor volumes in all treatment groups were still significantly smaller than those in the control group. Although no significant difference was observed between the cNP and anti-PD-1 monotherapy groups, both groups exhibited significantly larger tumor volumes compared with the cNP + anti-PD-1 group, indicating superior antitumor efficacy of the combination therapy. These results suggest that the enhanced antitumor effect of the combined treatment required approximately 1 week to become apparent. By day 22, significant differences in tumor volume were observed among all four groups. Collectively, these results demonstrate that cNP, anti-PD-1, and their combination all inhibited tumor progression, with the combined immunotherapy showing the strongest antitumor effect.

Survival analysis (Figure 4C) further supported these findings. The median survival times of mice in the control, cNP, and anti-PD-1 groups were 15, 30, and 33 days, respectively (n = 7). During the observation period (up to day 45), survival rates in the control, cNP, and anti-PD-1 groups declined to 0%, whereas the cNP + anti-PD-1 group maintained a survival rate of 71.4%. These results indicate that combination therapy not only suppressed tumor growth but also significantly prolonged the survival of tumor-bearing mice.

### 4.6 Combination therapy with cNP and anti-PD-1 significantly alleviated immunosuppression in the TME of HNSCC-bearing mice

Given the critical role of immunosuppression within the TME in limiting antitumor efficacy, immune cell infiltration in the TME was further evaluated.

**FIGURE 5:**
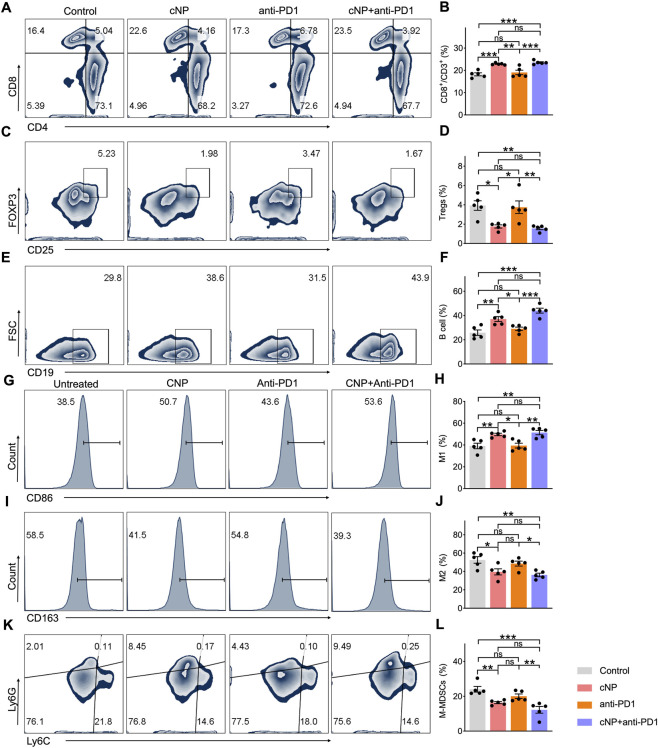
Combination therapy with cNP and anti-PD-1 significantly alleviated immunosuppression in the TME of HNSCC-bearing mice. Representative flow cytometry **(A,C,E,G,I,K)** and quantitative comparison of CD8^+^ T cells **(B)**, Treg cells **(D)**, CD19^+^ B cells **(F)**, M1 macrophages **(H)**, M2 macrophages **(J)**, M-MDSCs cells **(L)** in the tumor tissue of different treatment groups (n = 5, means ± s.d.; *0.01 < *P* < 0.05, **0.001 < *P* < 0.01, ***P < 0.001).

CD8^+^ T cells are key effector cells in antitumor immunity, directly eliminating tumor cells upon antigen recognition. Compared with the control group, the proportions of CD8^+^ T cells were significantly increased in the cNP and cNP + anti-PD-1 groups. Although the anti-PD-1 group also showed an increased proportion of CD8^+^ T cells, the difference was not statistically significant. Notably, the proportion of CD8^+^ T cells in the cNP + anti-PD-1 group was significantly higher than that in the anti-PD-1 group alone, indicating enhanced immune activation with combination therapy (Figures 5A,B).

As shown in Figures 5C,D, the proportion of regulatory T cells (Tregs) was significantly reduced in the cNP and cNP + anti-PD-1 groups compared with the control group. Moreover, Treg levels in the combination group were significantly lower than those in the anti-PD-1 group, suggesting that combination therapy effectively attenuated immunosuppression within the TME.

B cell proportions (Figures 5E,F) were significantly increased in the cNP and cNP + anti-PD-1 groups compared with the control group, with the combination group showing a significantly higher proportion than the anti-PD-1 group. Similarly, the proportion of M1 macrophages (Figures 5G,H) was significantly elevated in the cNP and combination groups, with higher levels observed in the combination group than in the anti-PD-1 group.

In contrast, the proportion of M2 macrophages (Figures 5I,J) was significantly decreased in the cNP and combination groups compared with the control group, and further reduced in the combination group compared with the anti-PD-1 group. M-MDSCs also showed a significant reduction in the cNP and combination groups (Figures 5K,L), with the lowest levels observed in the combination group.

Collectively, these results indicate that cNP combined with anti-PD-1 significantly enhanced both cellular and humoral immune responses by increasing CD8^+^ T cells and B cells. Moreover, the combination therapy promoted macrophage polarization from the pro-tumorigenic M2 phenotype toward the antitumor M1 phenotype and markedly reduced immunosuppressive cell populations, including Tregs and M-MDSCs. These immunomodulatory effects likely underlie the enhanced antitumor efficacy observed with combination therapy.

### 4.7 cNP decreases NETs infiltration in tumor tissue of HNSCC-bearing mice

**FIGURE 6:**
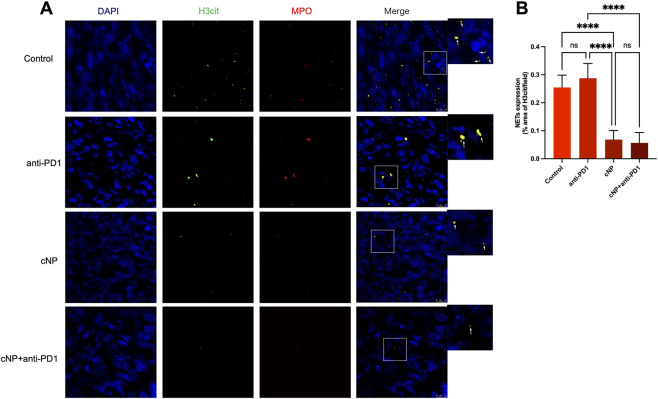
cNP decreases NETs infiltration in tumor tissue of HNSCC-bearing mice. **(A)** The expression of NETs in the tumor tissue of different treatment groups by using immunofluorescence staining for H3cit and MPO (scale bar: 10 μm). **(B)** NETs expression quantification demonstrated that cNP efficiently reduced NETs infiltration in tumor tissue (n = 5, means ± s.d.; ****P < 0.0001).

This study aimed to inhibit HNSCC progression by clearing NETs within tumor tissues. To assess the ability of cNP to eliminate NETs *in vivo*, tumor tissues from all four groups were collected on day 22 and subjected to immunofluorescence staining for MPO and H3Cit (Figure 6A). As shown in Figures 6A,B, compared with the control group, the anti-PD-1 group exhibited a slight elevation in NETs levels, although the difference was not statistically significant. In contrast, NETs expression was significantly decreased in both the cNP and cNP + anti-PD-1 groups compared with the control and anti-PD-1 groups. Combined with the aforementioned findings, these results suggest that clearance of NETs in HNSCC tissues enhances the therapeutic efficacy of anti-PD-1.

### 4.8 Effects of cNP on the viability, migration, cytoskeleton remodeling and adhesion of SCC9 cells

**FIGURE 7:**
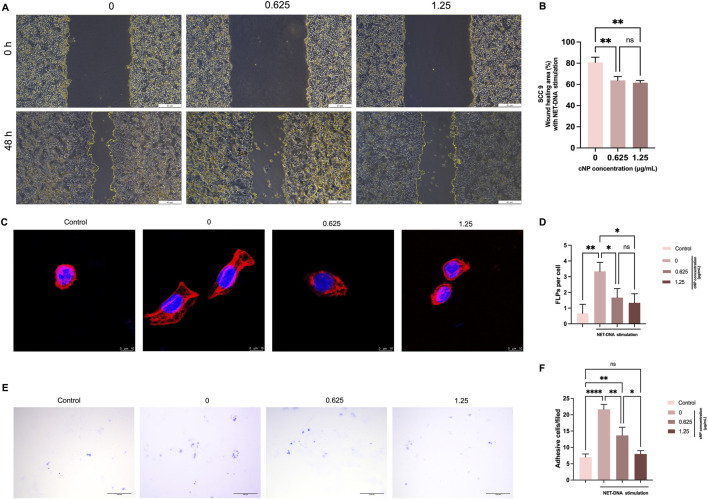
cNP inhibits chemotactic function of NET-DNA in HNSCC cells. **(A)** Representative images of SCC9 cells migration treated with cNP under NET-DNA stimulation, as assessed by wound healing assay. **(B)** Quantitative analysis of the wound area healing indicated cNP inhibited NET-DNA-induced migration of SCC9 cells (n = 3, means ± s.d.; **P < 0.01). **(C)** Confocal images of SCC9 cells treated with cNP, and then stained with phalloidin (F-actin, Red) and DAPI (Nuclei, Blue). **(D)** Quantification of FLPs showed that cNP restrained NET-DNA-induced cytoskeleton remodeling (n = 3, means ± s.d.; *0.01 < *P* < 0.05, **0.001 < *P* < 0.01). **(E)** Microscope images of SCC9 cells treated with cNP. **(F)** Quantification of adhesive SCC9 cells demonstrated cNP greater reduced the adhesion of SCC9 cells (n = 3, means ± s.d.; *0.01 < *P* < 0.05, **0.001 < *P* < 0.01, ****P < 0.0001).

To investigate the effect of NET-DNA on SCC9 cell migration, wound-healing assays were performed after 24 h of stimulation with different concentrations of NET-DNA (0, 0.5, 1, and 2 μg/mL) (Supplementary Figure S2A). As shown in Supplementary Figure S2B, NET-DNA significantly influenced SCC9 cell migration. Compared with the control group, migration was slightly increased at 0.5 μg/mL NET-DNA, although the difference was not statistically significant. At 1 μg/mL, SCC9 cell migration was significantly enhanced, whereas further increasing the concentration to 2 μg/mL resulted in a marked inhibition of migration. The selection of 1 μg/mL NET-DNA for subsequent experiments is based on dose-response data demonstrating maximal effect at this concentration. Under NET-DNA stimulation (1 μg/mL), the effects of different concentrations of cNP on SCC9 cell viability were evaluated (Supplementary Figure S3). The results showed that treatment with cNP (0.625 and 1.25 μg/mL) did not significantly affect SCC9 cell viability, indicating that cNP did not promote or inhibit cell proliferation under NET-DNA conditions. Wound-healing assays further demonstrated that under NET-DNA stimulation, low-dose cNP (0.625 μg/mL) significantly inhibited SCC9 cell migration compared with the only NET-DNA stimulation group (Figures 7A,B). Increasing the cNP concentration to 1.25 μg/mL did not result in additional inhibition, suggesting a plateau effect at higher concentrations. Cytoskeletal staining was performed to assess the effects of cNP on cytoskeletal remodeling under NET-DNA stimulation (Figure 7C), and the number of filopodia was quantitatively analyzed (Figure 7D). NET-DNA stimulation markedly increased filopodia formation compared with the control group. Treatment with low-dose cNP significantly reduced filopodia formation, whereas further increases in cNP concentration did not yield statistically significant additional effects. Cell adhesion assays revealed that NET-DNA significantly enhanced SCC9 cell adhesion, whereas cNP treatment reduced adhesion in a concentration-dependent manner (Figures 7E,F). These findings indicate that cNP effectively suppress NET-DNA induced migration, cytoskeletal remodeling, and adhesion of SCC9 cells. Overall, these results confirm that NET-DNA promotes HNSCC cell migration, adhesion, and cytoskeletal remodeling, potentially through interaction with the cell surface receptor CCDC25 (Yang et al., 2020). Moreover, cNP bind NET-DNA and inhibit these pro-metastatic effects in a partially concentration-dependent manner.

### 4.9 Expression of CCDC25 in HNSCC tissues and cell lines

**FIGURE 8:**
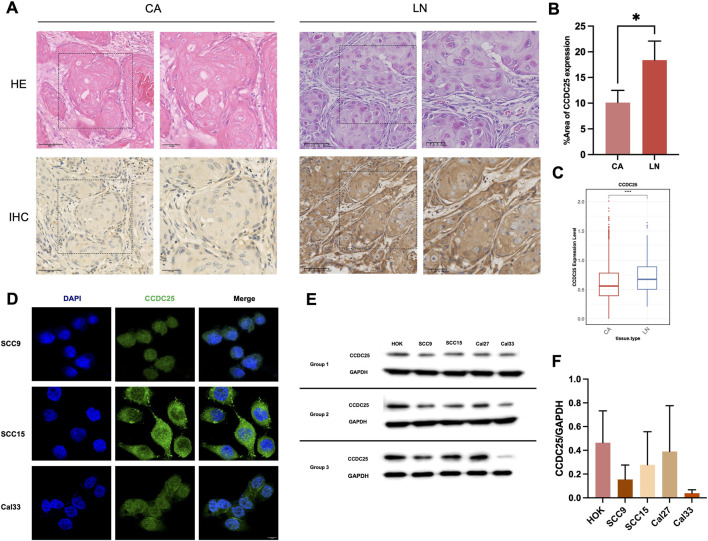
Expression of CCDC25 in HNSCC tissues and cell lines. **(A)** Representative HE, and IHC staining images of CCDC25 expression in primary cancer (CA) and lymph node metastasis (LN). **(B)** Quantification of IHC showed that CCDC25 was overexpressed in LA compared to CA (n = 3, means ± s.d.; *P < 0.05). **(C)** Single-cell RNA sequencing analysis of the GEO dataset (GSE181919) demonstrated that CCDC25 mRNA expression was significantly higher in LN than in CA samples (****P < 0.0001). **(D)** Representative immunofluorescence staining images of CCDC25 (Green) and DAPI (Blue) in HNSCC cell lines. **(E)** The protein level of CCDC25 was analyzed in Human Oral Keratinocytes (HOK) and HNSCC cell lines by WB. **(F)** Quantification of WB (n = 3).

Immunohistochemical staining was performed to compare CCDC25 expression in primary HNSCC lesions (CA) and metastatic lymph nodes (LN) (Figure 8A). Quantitative analysis revealed significantly higher CCDC25 expression in metastatic lesions compared with primary tumors (Figure 8B). Single-cell transcriptomic analysis of the GEO dataset (GSE181919) revealed that CCDC25 mRNA expression was generally low and broadly distributed in primary tumor samples, whereas LN samples exhibited a markedly higher median expression level (Figure 8C). These findings suggest a potential role of CCDC25 in lymph node metastasis of HNSCC. Immunofluorescence analysis demonstrated that CCDC25 protein was primarily localized to the cell membrane and cytoplasm in SCC9 and Cal33 cells, with minimal overlap with nuclear staining (Figure 8D), suggesting its potential involvement in NET-DNA binding at the cell surface. Western blot analysis further confirmed CCDC25 expression across multiple cell lines (HOK, SCC9, SCC15, Cal27, and Cal33), although expression levels varied (Figures 8E,F). CCDC25 expression was higher in HOK cells and generally downregulated in HNSCC cell lines, particularly in Cal33 cells, although differences were not statistically significant.

### 4.10 NET-DNA promotes HNSCC cell migration via CCDC25

**FIGURE 9:**
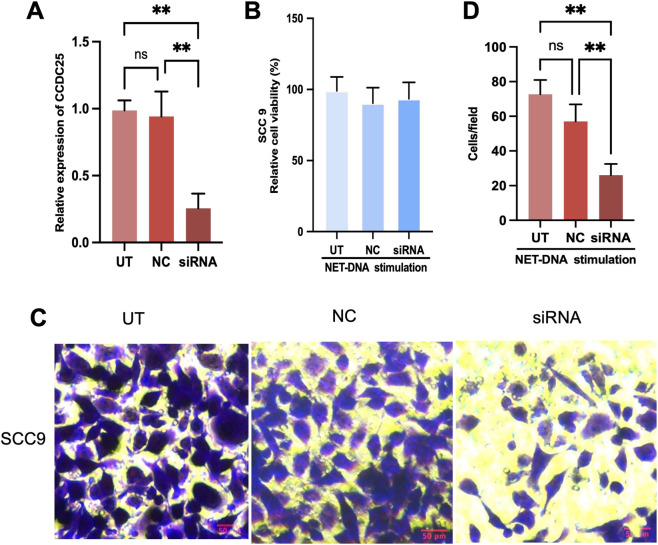
NET-DNA promotes HNSCC cell migration via CCDC25. **(A)** The mRNA expression of CCDC25 was determined by RT-qPCR in the UT (untreated), NC (negative control), and siRNA-CCDC25 groups (n = 3, means ± s.d.; **P < 0.01). **(B)** Cell viability of SCC9 with NET-DNA (1 μg/mL) stimulation was evaluated by CCK8 in different groups (n = 3, means ± s.d.). **(C)** Representative images from the transwell migration experiment assessing SCC9 cell migration upon stimulation with NET-DNA (1 μg/mL). **(D)** Quantification of cell migration demonstrated that NET-DNA promoted HNSCC cell migration through CCDC25 (n = 3, means ± s.d.; **P < 0.01).

To clarify the role of CCDC25 under NET-DNA stimulation, CCDC25 expression was silenced in SCC9 cells using siRNA, followed by NET-DNA treatment (1 μg/mL). qRT-PCR confirmed efficient knockdown of CCDC25 expression in the siRNA group compared with untreated (UT) and negative control (NC) groups (Figure 9A). CCK-8 assays showed no significant differences in cell viability among UT, NC, and siRNA groups under NET-DNA stimulation (Figure 9B), indicating that CCDC25 knockdown did not affect SCC9 cell proliferation. In contrast, transwell migration assays revealed a marked reduction in cell migration following CCDC25 silencing (Figures 9C,D). Compared with UT and NC groups, the siRNA group exhibited significantly fewer migrated cells. These results indicate that under NET-DNA stimulation, CCDC25 plays a critical role in regulating SCC9 cell migration but has minimal impact on cell proliferation.

### 4.11 Correlation analysis of CCDC25 and TIME of HNSCC

Bubble plot analysis revealed significant correlations between CCDC25 mRNA expression and multiple immune cell subsets based on CIBERSORT scores (Supplementary Figure S4A). CCDC25 expression was positively correlated with CD4^+^ memory resting T cells, CD4^+^ memory activated T cells, plasma B cells, and naïve B cells, while showing negative correlations with resting mast cells, M0 macrophages, and eosinophils.

Analysis of immune cell infiltration across different CCDC25 copy number variation states (Supplementary Figure S4B) showed that arm-level deletion of CCDC25 was associated with significantly reduced infiltration of B cells, CD8^+^ T cells, CD4^+^ T cells, and dendritic cells in overall HNSCC samples. Similar trends were observed in both HPV-negative and HPV-positive subgroups. Box plot analysis further demonstrated significantly higher CCDC25 expression in HPV-positive HNSCC compared with HPV-negative tumors (Supplementary Figure S4C). Scatter plot analysis revealed a significant positive correlation between CCDC25 expression and G2/M checkpoint pathway activity in HNSCC tissues (Supplementary Figure S4D). Additionally, CCDC25 expression was positively correlated with MPO expression (Supplementary Figure S4E). Combined with previous reports identifying CCDC25 as a NET-DNA receptor (Yang et al., 2020), these findings suggest that CCDC25 may function as a downstream mediator of NET-DNA signaling, facilitating tumor cell migration and metastasis in HNSCC.

## 5 Discussions

ICIs have demonstrated substantial therapeutic potential in the treatment of HNSCC (Kitamura et al., 2020). However, a subset of patients still exhibits limited therapeutic response or develops resistance. In addition, while ICIs enhance antitumor immune responses, they may also amplify systemic and local inflammatory reactions. Excessive immune activation can result in immune-related adverse events (irAEs), affecting multiple organs, including the skin, gastrointestinal tract, liver, lungs, and endocrine glands (Postow et al., 2018). More importantly, the sustained release of inflammatory cytokines, such as TNF-α, IL-6, and IFN-γ, not only disrupts tissue homeostasis but can also induce epithelial–mesenchymal transition (EMT) in tumor cells, remodel the TME, and thereby facilitate tumor invasion and distant metastasis (Elinav et al., 2013; Jiang and Zhan, 2020; Ruffe et al., 2015).

Accumulating evidence indicates that inflammatory factors within the TME are among the key determinants influencing the efficacy of PD-L1/PD-1 blockade therapy (Jiang et al., 2019). Therefore, investigations targeting the tumor microenvironment may contribute to improving the outcomes of PD-L1/PD-1 immunotherapy. NETs promote the formation of an immunosuppressive microenvironment through multiple mechanisms, thereby enhancing tumor immune evasion. In addition, NETs can form physical barriers and are closely associated with cancer metastasis (Yang et al., 2020; Liu et al., 2020; Milette et al., 2020). Accordingly, the clearance of NET-DNA represents a potential strategy to enhance the efficacy of immunotherapy in HNSCC. In this study, cNP was employed to clear NET-DNA and disrupt the associated physical barriers, thereby suppressing immune evasion of HNSCC cells. Moreover, the removal of NET-DNA inhibited NET-DNA–mediated cancer cell motility and metastasis, ultimately restraining HNSCC progression and improving the therapeutic efficacy of immunotherapy.

To investigate the role of NETs in HNSCC progression, we first assessed NETs expression in HNSCC tissues and adjacent non-tumor tissues. The results indicated that NETs were scarcely detected in adjacent tissues, whereas their expression was markedly elevated in tumor tissues. Consistent with previous reports demonstrating high NETs expression in various malignancies (Berger-Achituv et al., 2013; Demers and Wagner, 2013), these findings suggest that elevated NETs levels in HNSCC align with observations in other cancer types. To further elucidate the functional significance of NETs overexpression in HNSCC, this study focused on three aspects: proliferation, immune microenvironment modulation, and metastasis. Regarding tumor cell proliferation, CCK-8 assay results showed that within the tested concentration range (0, 0.5, 1, and 2 μg/mL), NETs exerted only mild effects on the proliferative capacity of four HNSCC cell lines (SCC9, SCC15, Cal27, and Cal33), without statistical significance. In line with previous studies (Masucci et al., 2020; Yan et al., 2023), these results suggest that NETs may promote tumor progression in HNSCC primarily by enhancing migration, invasion, and immune evasion rather than directly stimulating tumor cell proliferation.

Previous studies have reported that HNSCC is characterized by intratumoral regulatory T-cell infiltration, impaired natural killer cell function, an increased Treg/CD8^+^ T-cell ratio, and elevated expression of PD-L1 on tumor cells. Together, these factors contribute to a dysfunctional immune microenvironment that favors tumor growth, therapeutic resistance, and immune evasion (Wang et al., 2019). To explore the impact of NETs on the immune microenvironment of HNSCC, bioinformatics analyses were performed to evaluate the expression of the MPO gene and its associations with immune infiltration, copy number variation, and immune pathway activity. As shown in Figure 3A, MPO expression was significantly correlated with the infiltration levels of multiple immune cell types and was associated with weakened antitumor immune responses within the TME. Figure 3B further demonstrated that, compared with tumors harboring normal MPO copy numbers, HNSCC samples with arm-level gain of MPO exhibited significantly reduced infiltration of CD8^+^ T cells, CD4^+^ T cells, neutrophils, and dendritic cells, indicating that MPO copy number amplification suppresses immune cell infiltration and attenuates local antitumor immunity. Pearson correlation analysis further revealed a significant negative correlation between MPO expression and CD8^+^ T-cell levels. Collectively, these findings indicate that MPO is closely associated with multiple immune cell populations in HNSCC, and that high MPO expression may facilitate the establishment of an immunosuppressive microenvironment, thereby promoting immune evasion. Consequently, clearance of NETs and disruption of the physical barrier may effectively suppress immune escape in HNSCC.

Despite the promising efficacy of PD-L1/PD-1 immunotherapy in HNSCC, several limitations remain (Wu et al., 2022). To enhance therapeutic outcomes, we established a C3H tumor-bearing mouse model to evaluate the combined treatment of cNP and anti-PD-1 antibodies. The results demonstrated that, compared with the other three treatment groups, the combination therapy group exhibited the most pronounced tumor volume reduction and the strongest tumor-suppressive effect. Survival analysis further showed a significant extension of median survival in mice receiving cNP plus anti-PD-1 treatment. These findings indicate that combination therapy not only inhibits tumor growth but also significantly prolongs survival in HNSCC-bearing mice, outperforming either anti-PD-1 or cNP monotherapy. Flow cytometry analysis revealed significantly increased proportions of CD8^+^ T cells and B cells in the combination treatment group. CD8^+^ T cells are key effector cells in antitumor immunity and directly mediate tumor cell killing (Raskov et al., 2021). Concurrently, the proportions of immunosuppressive cells, including regulatory T cells, M2 macrophages, and M-MDSCs, were markedly reduced. In addition, cNP combined with anti-PD-1 promoted the polarization of macrophages from the M2 phenotype to the antitumor M1 phenotype (Boutilier and Elsawa, 2021). These results suggest that cNP combined with anti-PD-1 therapy remodel the tumor microenvironment, significantly reduce immunosuppression in HNSCC, and effectively suppress myeloid inflammation induced by PD-1 monotherapy. Immunofluorescence staining further demonstrated that MPO and H3Cit signals were significantly reduced in the cNP and cNP plus anti-PD-1 groups compared with the PBS and anti-PD-1 monotherapy groups, indicating effective clearance of NET-DNA by cNP.

The impact of NETs on HNSCC metastasis was further investigated. First, the biosafety of cNP was evaluated. CCK-8 assays confirmed that within the concentration range used in this study, cNP exhibited no significant cytotoxicity toward SCC9 cells, even in the presence of NET-DNA, demonstrating good biocompatibility. On this basis, we focused on the direct effects of NET-DNA on tumor cell migration. Wound-healing assays revealed that NET-DNA at a concentration of 1 μg/mL significantly enhanced the migratory capacity of SCC9 cells, suggesting that NET-DNA concentration within the TME may be a critical determinant of tumor cell migratory behavior. Accordingly, 1 μg/mL NET-DNA was selected as the optimal stimulation concentration for subsequent experiments.

Results from wound-healing assays, cytoskeletal remodeling analyses, and cell adhesion assays showed that under NET-DNA stimulation (1 μg/mL), low concentrations of cNP (0.625 μg/mL) significantly inhibited SCC9 cell migration, cytoskeletal remodeling, and adhesion, with inhibitory effects reaching saturation at higher concentrations (1.25 μg/mL). These findings indicate that cNP bind to NET-DNA and spatially block its interaction with cell surface receptors, thereby suppressing downstream signaling activation and ultimately inhibiting HNSCC cell migration, cytoskeletal reorganization, and adhesion. These results are consistent with our previous findings using nanoparticulate cationic poly (amino acids) to disrupt NETs and block cancer metastasis (Liang et al., 2023). Together, these data not only confirm the prometastatic potential of NET-DNA in HNSCC but also demonstrate that cNP effectively suppress NET-DNA–mediated metastatic effects.

To further elucidate the molecular mechanism underlying NET-DNA–induced HNSCC metastasis, we investigated its key receptor, CCDC25. Bioinformatics and immunohistochemical analyses revealed significantly higher CCDC25 expression in metastatic lesions compared with primary tumors, consistent with previous reports (Yang et al., 2020), suggesting a close association between CCDC25 and HNSCC metastasis. Increasing evidence indicates that CCDC25 is aberrantly upregulated in various malignancies. For example, in clear cell renal cell carcinoma (ccRCC), CCDC25 expression is significantly elevated in tumor tissues and cell lines compared with normal counterparts, and CCDC25 knockdown markedly suppresses cancer cell proliferation, migration, and invasion both *in vitro* and *in vivo* (Qian et al., 2024).

It is noteworthy that the seemingly contradictory the higher expression of CCDC25 in metastatic lesions (IHC) but lower expression in HNSCC cell lines (Western blot). This is an interesting observation that potentially reflects the complexity of protein regulation networks during tumor progression. This discrepancy may be due to the following reasons: Firstly, post-translational modifications may differ between HNSCC cell lines and solid tumor microenvironments. Secondly, tumor heterogeneity is also the key factor, IHC on tissue sections identify high expression in specific tumor cells within their native architecture, which includes heterogeneous populations, while Western blot on cell lines represent the expression from the homogeneous population. Finally, signals (e.g., hypoxia, cytokines, or stromal cells interactions) in the tumor microenvironment are crucial for maintaining the CCDC25 expression, but these signals are different *in vitro* culture conditions. Further experiments are needed to elucidate the specific regulatory mechanisms of CCDC25 in HNSCC. Immunofluorescence analysis further demonstrated that CCDC25 was primarily localized to the cell membrane and cytoplasm of HNSCC cells, providing direct morphological evidence supporting its role as a membrane receptor for NET-DNA. Subsequent experiments employing siRNA-mediated knockdown of CCDC25 in SCC9 cells revealed that under NET-DNA stimulation (1 μg/mL), CCDC25 silencing significantly reduced HNSCC cell migration without markedly affecting cell proliferation.These key findings confirm that CCDC25 is a critical receptor mediating NET-DNA–induced migratory signaling in HNSCC cells. It can therefore be inferred that CCDC25 recognizes and binds NET-derived DNA, activates migration-related signaling pathways, and promotes HNSCC metastasis, a process that appears largely independent of cell proliferation. Collectively, through bioinformatics analyses, validation using clinical tissue samples, and functional cellular assays, this study elucidates a crucial mechanism by which NET-DNA promotes HNSCC metastasis via its key receptor CCDC25, and demonstrates the effective intervention of this malignant process using cNP. These findings not only deepen our understanding of HNSCC metastasis but also identify CCDC25 as a potential therapeutic target and prognostic biomarker, providing a solid theoretical foundation for the development of anti-metastatic strategies targeting the NET-DNA-CCDC25 signaling axis. Finally, our findings demonstrate that cNP have the dual therapeutic effect by both clearing NET-DNA to remodel the tumor immune microenvironment and directly inhibiting the NET-DNA-CCDC25-mediated migration. Moreover, these two processes are likely interdependent and synergistic. On one hand, the clearance of NET-DNA by cNP removes a physical barrier to immune cell infiltration. This likely leads to long-term antitumor effect by improving cytotoxic T lymphocyte infiltration. On the other hand, the direct inhibition of CCDC25-mediated migration by cNP may provide an immediate blockade of tumor dissemination. Dissecting the dominant role of these mechanisms will be a key focus of our future investigations by using conditional knockout models or other methods.

From the perspectives of the immune microenvironment and metastasis in HNSCC, this study proposes a combined therapeutic strategy of “NET clearance plus immune checkpoint blockade” offering new insights and potential interventions for HNSCC immunotherapy, while acknowledging certain limitations.

While the sample size (direct tissue staining) for our clinical tissue validation is relatively small, the findings from our clinical tissue are further validated and consistent with the independent analysis of the larger GEO dataset. By integrating these two layers of evidence, these results could mitigate the limitations of the small cohort and strengthens the overall reliability of the conclusions. However, future studies with larger, independent clinical cohorts are necessary to confirm these findings. Although this study clarified that NET-DNA promotes HNSCC cell migration via CCDC25, the complete signaling networks through which NETs regulate immune evasion, tumor heterogeneity, and metastasis remain incompletely defined. Future studies integrating multi-omics approaches could systematically analyze interactions between NETs and immune cells within the TME, including CD8^+^ T cells, B cells, and macrophages, to identify key regulatory nodes involved in microenvironment remodeling, immune tolerance, and metastatic niche formation. Furthermore, our bioinformatics analysis revealed differences in immune infiltration profiles between HPV-positive and HPV-negative tumors, suggesting that HPV status may influence NET-related immune modulation in the tumor microenvironment; however, this finding falls outside the primary scope of this study and warrants further dedicated investigation. Finally, further investigation into the roles of NETs in angiogenesis, EMT, cell cycle regulation, and copy number variation in HNSCC may help construct a more comprehensive molecular regulatory network to support precise therapeutic interventions.

In terms of preclinical validation and safety assessment, the present study was primarily based on tumor-bearing mouse models. Although cNP demonstrated favorable antitumor efficacy and immune-modulatory capacity *in vivo*, their performance in models more closely resembling clinical conditions has not yet been systematically evaluated. Therefore, future studies should assess the safety, targeting specificity, pharmacokinetics, and immunoregulatory effects of cNP in patient-derived organoids (PDOs) and other clinically relevant models to facilitate translational application.Furthermore, beyond NET-DNA clearance, cNP also improve immunosuppressive microenvironments, highlighting their considerable potential in combination therapies. Future investigations may explore synergistic effects between cNP and radiotherapy, chemotherapy, targeted therapies, or other immunotherapeutic modalities to achieve enhanced antitumor activity. Meanwhile, optimization of cNP size, surface charge, degradability, and biocompatibility may further improve tumor targeting, prolong *in vivo* retention, and reduce potential adverse effects. Integration of stimuli-responsive nanomaterials, such as pH-, enzyme-, or ROS-responsive systems, may enable controlled release and targeted clearance, further enhancing therapeutic precision and clinical translatability.Finally, based on the proposed “NETs–immune checkpoint–metastasis” integrated regulatory model, future preclinical studies may focus on personalized therapeutic strategies that incorporate tumor heterogeneity assessment and immune status prediction, thereby providing precision immunotherapy for HNSCC patients. This model may also offer theoretical guidance and potential intervention strategies for overcoming immunotherapy resistance and metastasis in other solid tumors.

## 6 Conclusion

Focusing on the central concept of “NETs–immune checkpoint–metastasis,” this study systematically elucidates the critical roles of NETs in promoting immune evasion and metastasis in HNSCC. In particular, the molecular mechanism and significance of the NET-DNA–CCDC25 signaling axis in HNSCC metastasis are comprehensively revealed. Moreover, cNP effectively inhibit immune evasion and metastasis in HNSCC by efficiently clearing NET-DNA. A combined therapeutic strategy involving cNP and anti-PD-1 antibodies was further established, which remodels the immune microenvironment, enhances T cell–mediated antitumor responses, and significantly improves the overall efficacy of immunotherapy. By proposing a novel “NETs–immune checkpoint–metastasis” integrated therapeutic paradigm, this study provides a new theoretical framework and potential translational direction for addressing immunotherapy resistance and metastasis in HNSCC, with substantial scientific significance and promising clinical prospects.

## Data Availability

The datasets presented in this study can be found in online repositories. The names of the repository/repositories and accession number(s) can be found in the article/Supplementary Material.

## References

[B1] Berger-Achituv S. Brinkmann V. Abed U. A. Kühn L. I. Ben-Ezra J. Elhasid R. (2013). A proposed role for neutrophil extracellular traps in cancer immunoediting. Front. Immunol. 4, 48. 10.3389/fimmu.2013.00048 23508552 PMC3589747

[B2] Bhatia A. Burtness B. (2023). Treating head and neck cancer in the age of immunotherapy: a 2023 update. Drugs 83 (3), 217–248. 10.1007/s40265-023-01835-2 36645621

[B3] Boutilier A. J. Elsawa S. F. (2021). Macrophage polarization states in the tumor microenvironment. Int. J. Mol. Sci. 22 (13), 6995. 10.3390/ijms22136995 34209703 PMC8268869

[B4] Bronte V. Brandau S. Chen S. H. Colombo M. P. Frey A. B. Greten T. F. (2016). Recommendations for myeloid-derived suppressor cell nomenclature and characterization standards. Nat. Communications 7 (1), 12150. 10.1038/ncomms12150 27381735 PMC4935811

[B5] Choi J.-H. Lee B. S. Jang J. Y. Lee Y. S. Kim H. J. Roh J. (2023). Single-cell transcriptome profiling of the stepwise progression of head and neck cancer. Nat. Commun. 14 (1), 1055. 10.1038/s41467-023-36691-x 36828832 PMC9958029

[B6] Cohen E. E. Bell R. B. Bifulco C. B. Burtness B. Gillison M. L. Harrington K. J. (2019). The society for immunotherapy of cancer consensus statement on immunotherapy for the treatment of squamous cell carcinoma of the head and neck (HNSCC). J. Immunotherapy Cancer 7, 1–31. 10.1186/s40425-019-0662-5 31307547 PMC6632213

[B7] Demers M. Wagner D. D. (2013). Neutrophil extracellular traps: a new link to cancer-associated thrombosis and potential implications for tumor progression. Oncoimmunology 2 (2), e22946. 10.4161/onci.22946 23526174 PMC3601165

[B8] Denaro N. Merlano M. C. Lo Nigro C. (2021). Further understanding of the immune microenvironment in head and neck squamous cell carcinoma: implications for prognosis. Cancer Manag. Res. 13, 3973–3980. 10.2147/CMAR.S277907 34040438 PMC8139676

[B9] Elinav E. Nowarski R. Thaiss C. A. Hu B. Jin C. Flavell R. A. (2013). Inflammation-induced cancer: crosstalk between tumours, immune cells and microorganisms. Nat. Rev. Cancer 13 (11), 759–771. 10.1038/nrc3611 24154716

[B10] Fang Q. Stehr A. M. Naschberger E. Knopf J. Herrmann M. Stürzl M. (2022). No NETs no TIME: crosstalk between neutrophil extracellular traps and the tumor immune microenvironment. Front. Immunology 13, 1075260. 10.3389/fimmu.2022.1075260 36618417 PMC9816414

[B11] Gao L. Zhang W. Zhong W. Q. Liu Z. J. Li H. M. Yu Z. L. (2018). Tumor associated macrophages induce epithelial to mesenchymal transition *via* the EGFR/ERK1/2 pathway in head and neck squamous cell carcinoma. Oncol. Reports 40 (5), 2558–2572. 10.3892/or.2018.6657 30132555 PMC6151899

[B12] Hong Q. Ding S. Xing C. Mu Z. (2024). Advances in tumor immune microenvironment of head and neck squamous cell carcinoma: a review of literature. Medicine 103 (9), e37387. 10.1097/MD.0000000000037387 38428879 PMC10906580

[B13] Jayaraman P. Parikh F. Newton J. M. Hanoteau A. Rivas C. Krupar R. (2018). TGF-β1 programmed myeloid-derived suppressor cells (MDSC) acquire immune-stimulating and tumor killing activity capable of rejecting established tumors in combination with radiotherapy. Oncoimmunology 7 (10), e1490853. 10.1080/2162402X.2018.1490853 30288358 PMC6169570

[B14] Jiang Y. Zhan H. (2020). Communication between EMT and PD-L1 signaling: new insights into tumor immune evasion. Cancer Lett. 468, 72–81. 10.1016/j.canlet.2019.10.013 31605776

[B15] Jiang X. Wang J. Deng X. Xiong F. Ge J. Xiang B. (2019). Role of the tumor microenvironment in PD-L1/PD-1-mediated. Mol. Cancer 18 (1), 10. 10.1186/s12943-018-0928-4 30646912 PMC6332843

[B16] Jie H. Gildener-Leapman N. Li J. Srivastava R. M. Gibson S. P. Whiteside T. L. (2013). Intratumoral regulatory T cells upregulate immunosuppressive molecules in head and neck cancer patients. Br. Journal Cancer 109 (10), 2629–2635. 10.1038/bjc.2013.645 24169351 PMC3833228

[B17] Jimenez L. Jayakar S. K. Ow T. J. Segall J. E. (2015). Mechanisms of invasion in head and neck cancer. Archives Pathology and Laboratory Medicine 139 (11), 1334–1348. 10.5858/arpa.2014-0498-RA 26046491 PMC7469951

[B18] Kaltenmeier C. Yazdani H. O. Morder K. Geller D. A. Simmons R. L. Tohme S. (2021). Neutrophil extracellular traps promote T cell exhaustion in the tumor microenvironment. Front. Immunology 12, 785222. 10.3389/fimmu.2021.785222 34899751 PMC8652262

[B19] Keskinov A. A. Shurin M. R. (2015). Myeloid regulatory cells in tumor spreading and metastasis. Immunobiology 220 (2), 236–242. 10.1016/j.imbio.2014.07.017 25178934

[B20] Kitamura N. Sento S. Yoshizawa Y. Sasabe E. Kudo Y. Yamamoto T. (2020). Current trends and future prospects of molecular targeted therapy in head and neck squamous cell carcinoma. Int. Journal Molecular Sciences 22 (1), 240. 10.3390/ijms22010240 33383632 PMC7795499

[B21] Liang H. Du Y. Zhu C. Zhang Z. Liao G. Liu L. (2023). Nanoparticulate cationic poly (amino acid) s block cancer metastases by destructing neutrophil extracellular traps. ACS Nano 17 (3), 2868–2880. 10.1021/acsnano.2c11280 36648411

[B22] Liberzon A. Birger C. Thorvaldsdóttir H. Ghandi M. Mesirov J. P. Tamayo P. (2015). The Molecular Signatures Database (MSigDB) hallmark gene set collection. Cell Syst. 1 (6), 417–425. 10.1016/j.cels.2015.12.004 26771021 PMC4707969

[B23] Liu R. Zhao E. Wang F. Cui H. (2020). CCDC25: precise navigator for neutrophil extracellular traps on the prometastatic road. Signal Transduct. Target Ther. 5 (1), 162. 10.1038/s41392-020-00285-6 32839429 PMC7445241

[B24] Liu X. Liang H. Yan Y. Wu J. Bottini M. Liu L. (2022). The protein Corona modulates the inflammation inhibition by cationic nanoparticles *via* cell-free DNA scavenging. Bioact. Mater. 13, 249–259. 10.1016/j.bioactmat.2021.10.044 35224306 PMC8843952

[B25] Luginbuhl A. J. Johnson J. M. Harshyne L. A. Linnenbach A. J. Shukla S. K. Alnemri A. (2022). Tadalafil enhances immune signatures in response to neoadjuvant nivolumab in resectable head and neck squamous cell carcinoma. Clin. Cancer Res. 28 (5), 915–927. 10.1158/1078-0432.CCR-21-1816 34911681 PMC8898272

[B26] Masucci M. T. Minopoli M. Del Vecchio S. Carriero M. V. (2020). The emerging role of neutrophil extracellular traps (NETs) in tumor progression and metastasis. Front. Immunol. 11, 1749. 10.3389/fimmu.2020.01749 33042107 PMC7524869

[B27] Milette S. Quail D. F. Spicer J. D. (2020). Neutrophil DNA webs untangled. Cancer Cell 38 (2), 164–166. 10.1016/j.ccell.2020.07.002 32781042

[B28] Miraki Feriz A. Bahraini F. Khosrojerdi A. Azarkar S. Sajjadi S. M. HosseiniGol E. (2023). Deciphering the immune landscape of head and neck squamous cell carcinoma: a single-cell transcriptomic analysis of regulatory T cell responses to PD-1 blockade therapy. Plos One 18 (12), e0295863. 10.1371/journal.pone.0295863 38096229 PMC10721039

[B29] Nakazawa D. Shida H. Kusunoki Y. Miyoshi A. Nishio S. Tomaru U. (2016). The responses of macrophages in interaction with neutrophils that undergo NETosis. J. Autoimmun. 67, 19–28. 10.1016/j.jaut.2015.08.018 26347075

[B30] Newman A. M. Liu C. L. Green M. R. Gentles A. J. Feng W. Xu Y. (2015). Robust enumeration of cell subsets from tissue expression profiles. Nat. Methods 12 (5), 453–457. 10.1038/nmeth.3337 25822800 PMC4739640

[B31] Noubouossie D. F. Whelihan M. F. Yu Y. B. Sparkenbaugh E. Pawlinski R. Monroe D. M. (2017). *In vitro* activation of coagulation by human neutrophil DNA and histone proteins but not neutrophil extracellular traps. Blood, J. Am. Soc. Hematol. 129 (8), 1021–1029. 10.1182/blood-2016-06-722298 27919911 PMC5324715

[B32] Ou D. Adam J. Garberis I. Blanchard P. Nguyen F. Levy A. (2017). Clinical relevance of tumor infiltrating lymphocytes, PD-L1 expression and correlation with HPV/p16 in head and neck cancer treated with bio-or chemo-radiotherapy. Oncoimmunology 6 (9), e1341030. 10.1080/2162402X.2017.1341030 28932643 PMC5599076

[B33] Park J. Wysocki R. W. Amoozgar Z. Maiorino L. Fein M. R. Jorns J. (2016). Cancer cells induce metastasis-supporting neutrophil extracellular DNA traps. Sci. Translational Medicine 8 (361), 361ra138. 10.1126/scitranslmed.aag1711 27798263 PMC5550900

[B34] Postow M. A. Sidlow R. Hellmann M. D. (2018). Immune-related adverse events associated with immune checkpoint blockade. N. Engl. J. Med. 378 (2), 158–168. 10.1056/NEJMra1703481 29320654

[B35] Qian Z. Zhao H. Zhang Y. Wang Z. Zeng F. Zhu Y. (2024). Coiled-coil domain containing 25 (CCDC25) regulates cell proliferation, migration, and invasion in clear cell renal cell carcinoma by targeting the ILK-NF-κB signaling pathway. Faseb J. 38 (2), e23414. 10.1096/fj.202301064RR 38236371

[B36] Raskov H. Orhan A. Christensen J. P. Gögenur I. (2021). Cytotoxic CD8+ T cells in cancer and cancer immunotherapy. Br. Journal Cancer 124 (2), 359–367. 10.1038/s41416-020-01048-4 32929195 PMC7853123

[B37] Raskov H. Orhan A. Gaggar S. Gögenur I. (2022). Neutrophils and polymorphonuclear myeloid-derived suppressor cells: an emerging battleground in cancer therapy. Oncogenesis 11 (1), 22. 10.1038/s41389-022-00398-3 35504900 PMC9065109

[B38] Riquelme P. (2020). Exploring the relationship between circulating M-MDSC numbers and risk of posttransplant malignancy. Transplantation 104 (12), 2475–2477. 10.1097/TP.0000000000003180 32058464

[B39] Ruffell B. Coussens L. M. (2015). Macrophages and therapeutic resistance in cancer. Cancer Cell 27 (4), 462–472. 10.1016/j.ccell.2015.02.015 25858805 PMC4400235

[B40] Rutihinda C. Haroun R. Saidi N. E. Ordoñez J. P. Naasri S. Lévesque D. (2023). Inhibition of the CCR6-CCL20 axis prevents regulatory T cell recruitment and sensitizes head and neck squamous cell carcinoma to radiation therapy. Cancer Immunol. Immunother. 72 (5), 1089–1102. 10.1007/s00262-022-03313-2 36326893 PMC10991657

[B41] She L. Qin Y. Wang J. Liu C. Zhu G. Li G. (2018). Tumor-associated macrophages derived CCL18 promotes metastasis in squamous cell carcinoma of the head and neck. Cancer Cell International 18, 1–14. 10.1186/s12935-018-0620-1 30181713 PMC6114178

[B42] Siegel R. L. Kratzer T. B. Giaquinto A. N. Sung H. Jemal A. (2025). Cancer statistics, 2025. Cancer Stat. 75 (1), 10–45. 10.3322/caac.21871 39817679 PMC11745215

[B43] Solito S. Pinton L. Damuzzo V. Mandruzzato S. (2012). Highlights on molecular mechanisms of MDSC-Mediated immune suppression: paving the way for new working hypotheses. Immunol. Investigations 41 (6-7), 722–737. 10.3109/08820139.2012.678023 23017143

[B44] Song M. Zhang C. Cheng S. Ouyang D. Ping Y. Yang J. (2024). DNA of neutrophil extracellular traps binds TMCO6 to impair CD8+ T-cell immunity in hepatocellular carcinoma. Cancer Res. 84 (10), 1613–1629. 10.1158/0008-5472.CAN-23-2986 38381538

[B45] Veglia F. Sanseviero E. Gabrilovich D. I. (2021). Myeloid-derived suppressor cells in the era of increasing myeloid cell diversity. Nat. Rev. Immunol. 21 (8), 485–498. 10.1038/s41577-020-00490-y 33526920 PMC7849958

[B46] Wang Y. Krieg A. M. (2003). Synergy between cpg‐or non‐cpg DNA and specific antigen for B cell activation. Int. Immunology 15 (2), 223–231. 10.1093/intimm/dxg020 12578852

[B47] Wang H. C. Chan L. P. Cho S. F. (2019). Targeting the immune microenvironment in the treatment of head and neck squamous cell carcinoma. Front. Oncol. 9, 1084. 10.3389/fonc.2019.01084 31681613 PMC6803444

[B48] Wang H. Zhang H. Wang Y. Brown Z. J. Xia Y. Huang Z. (2021). Regulatory T-cell and neutrophil extracellular trap interaction contributes to carcinogenesis in non-alcoholic steatohepatitis. J. Hepatology 75 (6), 1271–1283. 10.1016/j.jhep.2021.07.032 34363921 PMC12888775

[B49] Warnatsch A. Ioannou M. Wang Q. Papayannopoulos V. (2015). Neutrophil extracellular traps license macrophages for cytokine production in atherosclerosis. Science 349 (6245), 316–320. 10.1126/science.aaa8064 26185250 PMC4854322

[B50] Wei C. Yang C. Wang S. Shi D. Zhang C. Lin X. (2019). Crosstalk between cancer cells and tumor associated macrophages is required for mesenchymal circulating tumor cell-mediated colorectal cancer metastasis. Mol. Cancer 18, 1–23. 10.1186/s12943-019-0976-4 30927925 PMC6441214

[B51] Wu K. Mao Y. Y. Han N. N. Wu H. Zhang S. (2021). PLAU1 facilitated proliferation, invasion, and metastasis *via* interaction with MMP1 in head and neck squamous carcinoma. Front. Oncol. 11, 574260. 10.3389/fonc.2021.574260 33816223 PMC8013724

[B52] Wu M. Huang Q. Xie Y. Wu X. Ma H. Zhang Y. (2022). Improvement of the anticancer efficacy of PD-1/PD-L1 blockade *via* combination therapy and PD-L1 regulation. J. Hematol. Oncol. 15 (1), 24. 10.1186/s13045-022-01242-2 35279217 PMC8917703

[B53] Yan M. Gu Y. Sun H. Ge Q. (2023). Neutrophil extracellular traps in tumor progression and immunotherapy. Front. Immunol. 14, 1135086. 10.3389/fimmu.2023.1135086 36993957 PMC10040667

[B54] Yang L. Liu Q. Zhang X. Liu X. Zhou B. Chen J. (2020). DNA of neutrophil extracellular traps promotes cancer metastasis *via* CCDC25. Nature 583 (7814), 133–138. 10.1038/s41586-020-2394-6 32528174

[B55] Zhang L. Yi H. Chen J. Li H. Luo Y. Cheng T. (2022). Neutrophil extracellular traps facilitate A549 cell invasion and migration in a macrophage‐maintained inflammatory microenvironment. BioMed Res. Int. 2022 (1), 8316525. 10.1155/2022/8316525 35036439 PMC8758275

[B56] Zhou S.-L. Zhou Z. J. Hu Z. Q. Huang X. W. Wang Z. Chen E. B. (2016). Tumor-associated neutrophils recruit macrophages and T-regulatory cells to promote progression of hepatocellular carcinoma and resistance to sorafenib. Gastroenterology 150 (7), 1646–1658. 10.1053/j.gastro.2016.02.040 26924089

