# Supplementary material for: Cationic nanoparticles with disrupting neutrophil extracellular traps inhibit the progression of head and neck squamous cell carcinoma
Source: Front Cell Dev Biol. 2026 Apr 22;14:1803439. doi: 10.3389/fcell.2026.1803439 (PMC13143943; doi:10.3389/fcell.2026.1803439)
Supplement: Supplementary file 1 [file Table1.doc]

| Gene | Sequence（5’-3’） |
| --- | --- |
| CCDC25-1 | GAGAGAAUAUAGAAGACAUdTdT |
|  | AUGUCUUCUAUAUUCUCUCdTdT |
| CCDC25-2 | GGAUCAUGGUGUUCUACUUdTdT |
|  | AAGUAGAACACCAUGAUCCdTdT |
| CCDC25-3 | GAUCUGAUCAAGCAUGGCUdTdT |
|  | AGCCAUGCUUGAUCAGAUCdTdT |
| NC | UUCUCCGAACGUGUCACGUdTdT |
|  | ACGUGACACGUUCGGAGAAdTdT |
| Fam NC | UUCUCCGAACGUGUCACGUdTdT |
|  | ACGUGACACGUUCGGAGAAdTdT |
| GAPDH | GUGGAGAUUGUUGCCAUCAdTdT |
|  | UGAUGGCAACAAUCUCCACdTdT |

Supplementary Table 1 siRNA sequence
