# Supplementary material for: Cationic nanoparticles with disrupting neutrophil extracellular traps inhibit the progression of head and neck squamous cell carcinoma
Source: Front Cell Dev Biol. 2026 Apr 22;14:1803439. doi: 10.3389/fcell.2026.1803439 (PMC13143943; doi:10.3389/fcell.2026.1803439)
Supplement: Supplementary file 2 [file Table2.docx]

| Genes | Sequence (5’-3’) |
| --- | --- |
| *CCDC25* | Forward primer: TGACAGTGGAGAAGAAAGTAAATGA  Reverse primer: CATTGCCATCCTGATTTGAAGACA |
| *GAPDH* | Forward primer: ACTCCTCCACCTTTGACGCT  Reverse primer: GGTCTCTCTCTTCCTCTTGTGC |

Supplementary Table 2. Primers used for qRT-PCR analysis
