# Supplementary material for: Cationic nanoparticles with disrupting neutrophil extracellular traps inhibit the progression of head and neck squamous cell carcinoma
Source: Front Cell Dev Biol. 2026 Apr 22;14:1803439. doi: 10.3389/fcell.2026.1803439 (PMC13143943; doi:10.3389/fcell.2026.1803439)
Supplement: Supplementary file 3 [file DataSheet1.docx]

**Cationic nanoparticles with disrupting neutrophil extracellular traps inhibit the progression of head and neck squamous cell carcinoma**

Zhaoqiang Zhang ^1#^, Yujie Kang ^2^, Bingxu Lu ^2^, Guichao Zhang ^2^, Baohan Xie ^2^, Yunyi Wang ^2^

^1^ School of Materials Science and Engineering, Key Laboratory for Polymeric Composite and Functional Materials of Ministry of Education, Sun Yat-sen University, Guangzhou 510275, P. R. China.

^2^ Department of Oral and Maxillofacial Surgery, Stomatological Hospital, School of Stomtology, Southern Medical University, Guangzhou 510280, P. R. China.

^#^ Corresponding authors:

Zhaoqiang Zhang (zhzhaoq2@mail3.sysu.edu.cn; [187234415@qq.com](mailto:187234415@qq.com)).


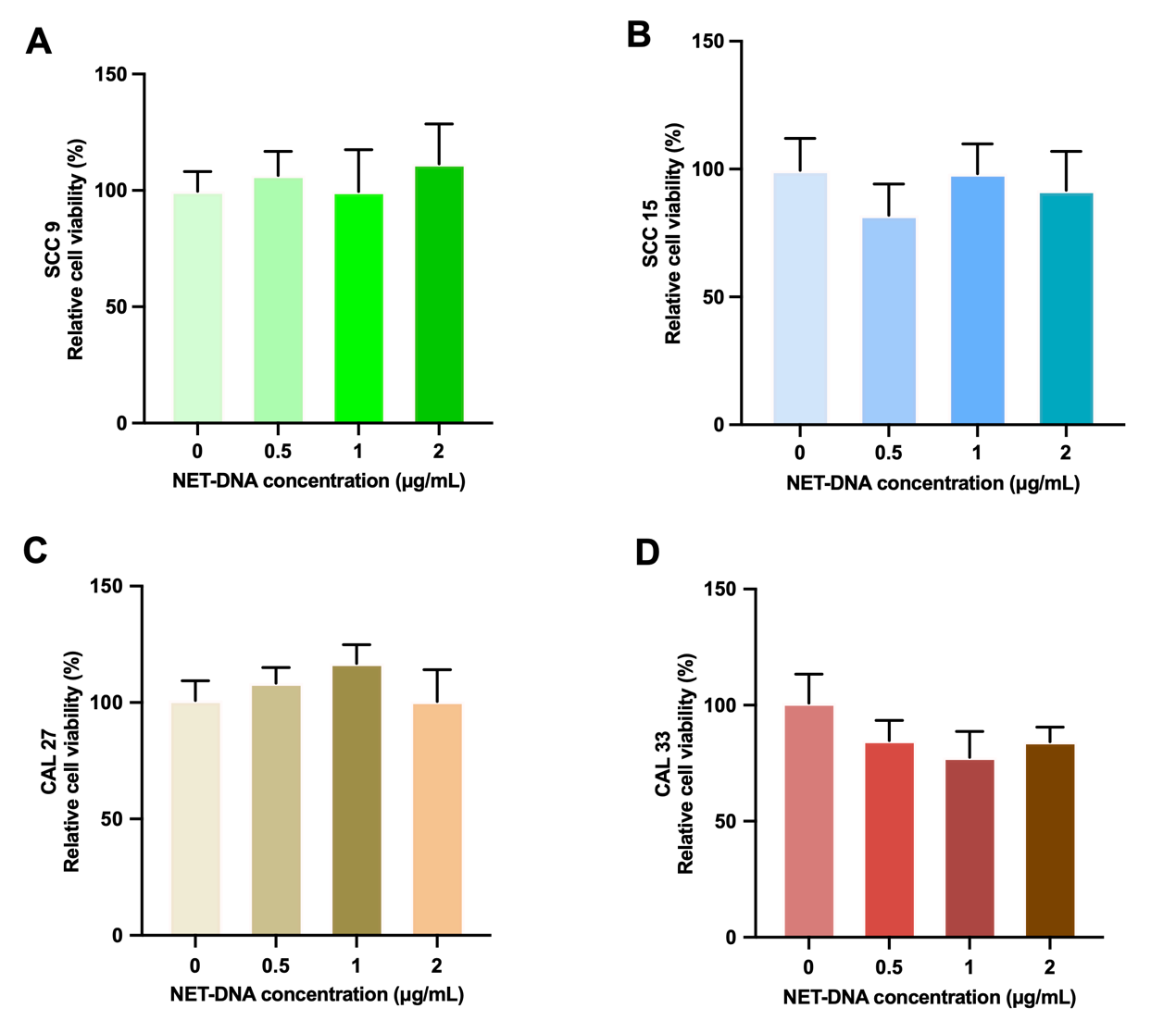


**Figure S1.** Effects of different concentrations of NET-DNA (0, 0.5, 1, 2 μg/ml) on the cell viability of four HNSCC cell lines: (A) SCC9; (B) SCC15; (C) Cal27 and (D) Cal33. (n = 3, means ± s.d.).


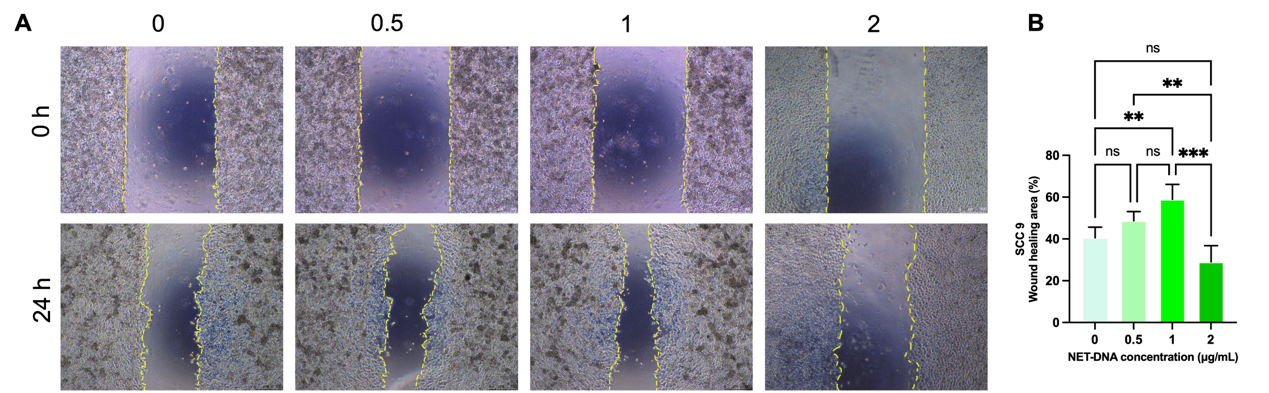


**Figure S2.** NET-DNA promoted SCC9 cells migration. (A) Representative images of SCC9 cells migration treated with NET-DNA (0, 0.5, 1.0, 2.0 μg/mL) stimulation, as evaluated using the wound healing assay. (B) Quantitative analysis of the wound area healing indicated NET-DNA promoted SCC9 cell migration (n = 3, means ± s.d.; **0.001 < *P* < 0.01, ****P* < 0.001).


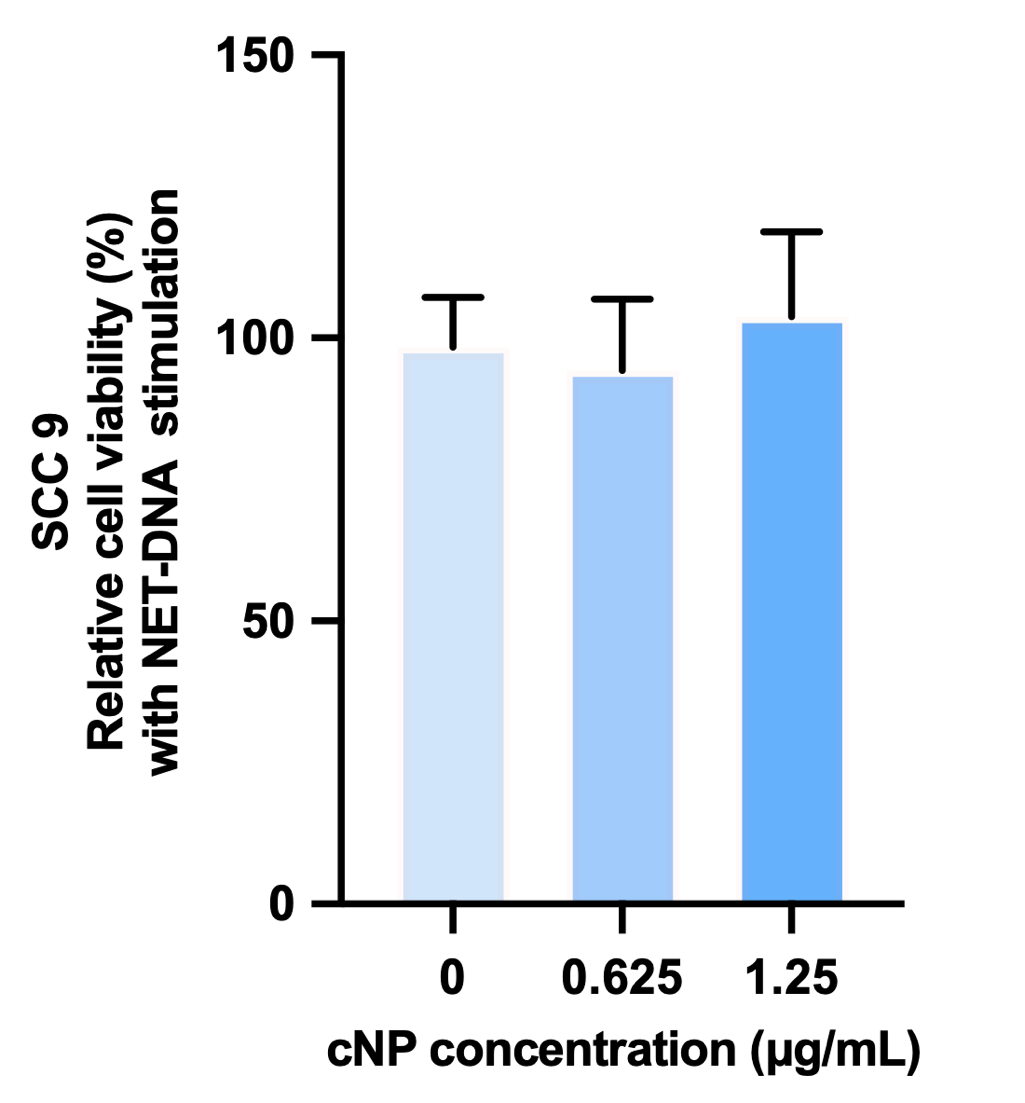


**Figure S3.** Evaluation of the effects of cNP on the viability of SCC9 cells with NET-DNA (1 μg/mL) stimulation by CCK8 asasy (n = 3, means ± s.d.).


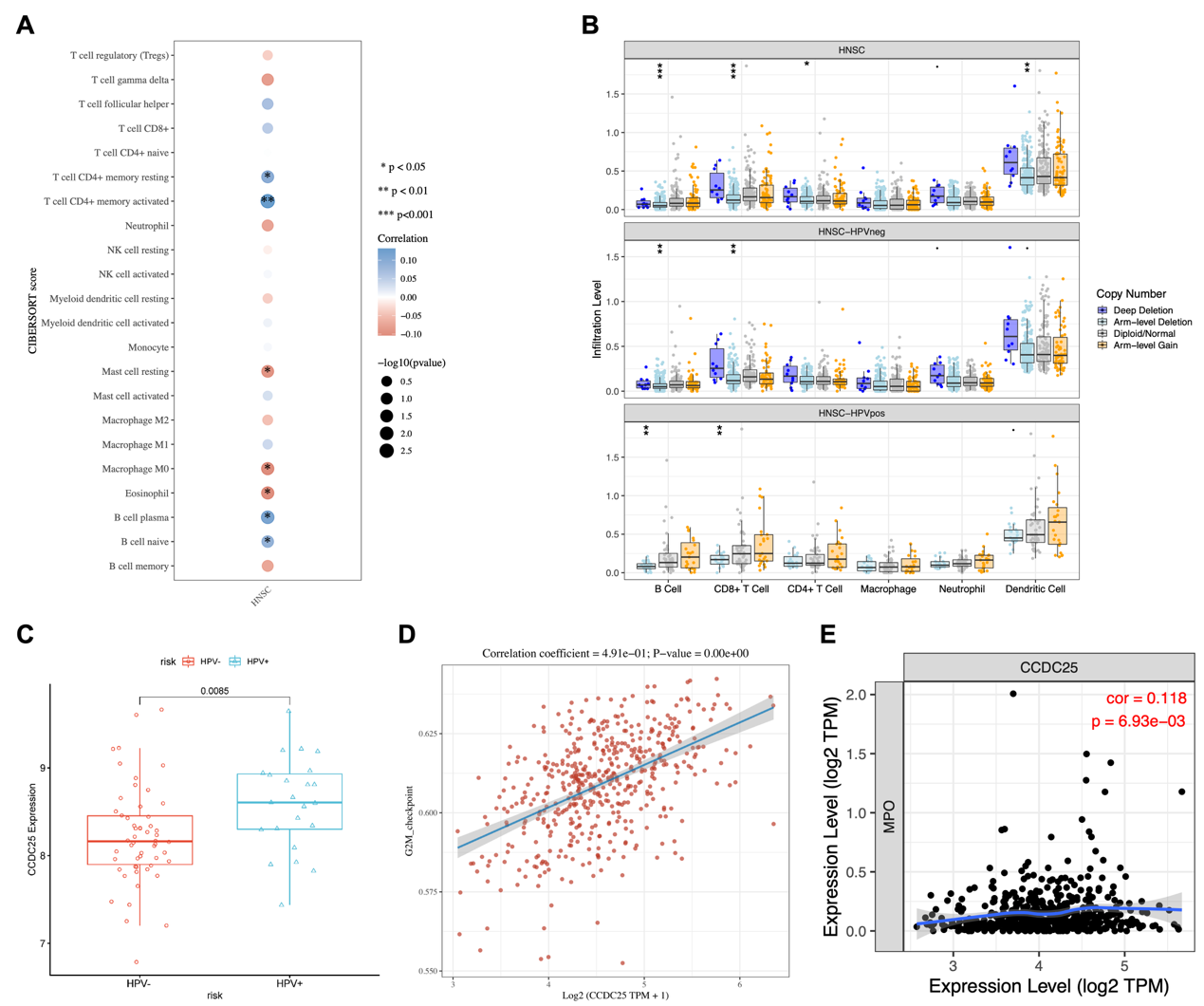


**Figure S4.** Correlation analysis of CCDC25 and TIME of HNSCC. (A) The expression level of CCDC25 is significantly correlated with the infiltration degree of various immune cells (*0.01 < *P* < 0.05, **0.001 < *P* < 0.01). (B) The infiltration levels of immune cells in the TIME of HNSCC under different copy number variation states of CCDC25 (*0.01 < *P* < 0.05, **0.001 < *P* < 0.01, ****P* < 0.001). (C) Expression of CCDC25 in HPV^+^ and HPV^-^ subtypes (*P* < 0.01). Correlation analysis of CCDC25 expression and G2/M checkpoint (D, *P* < 0.0001) and MPO (E, *P* < 0.01) in HNSCC.


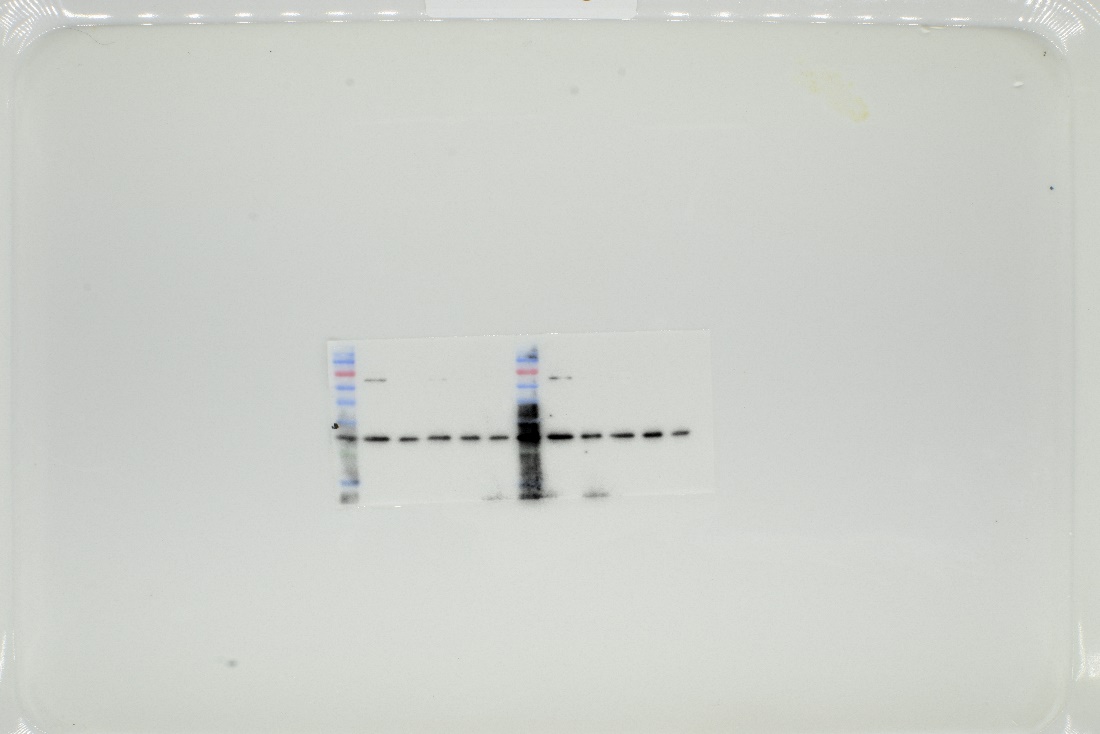


**Figure S5.** Full scan of the entire original gel (CCDC 25: Group 1 and 2).


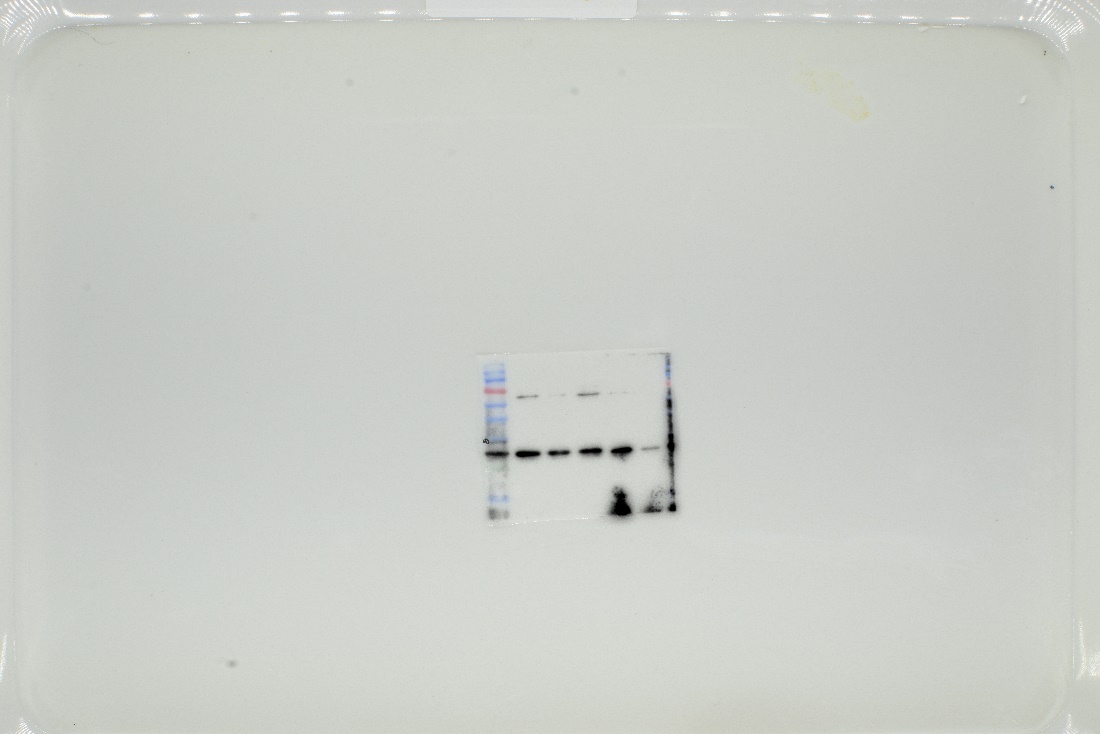


**Figure S6.** Full scan of the entire original gel (CCDC 25: Group 3).


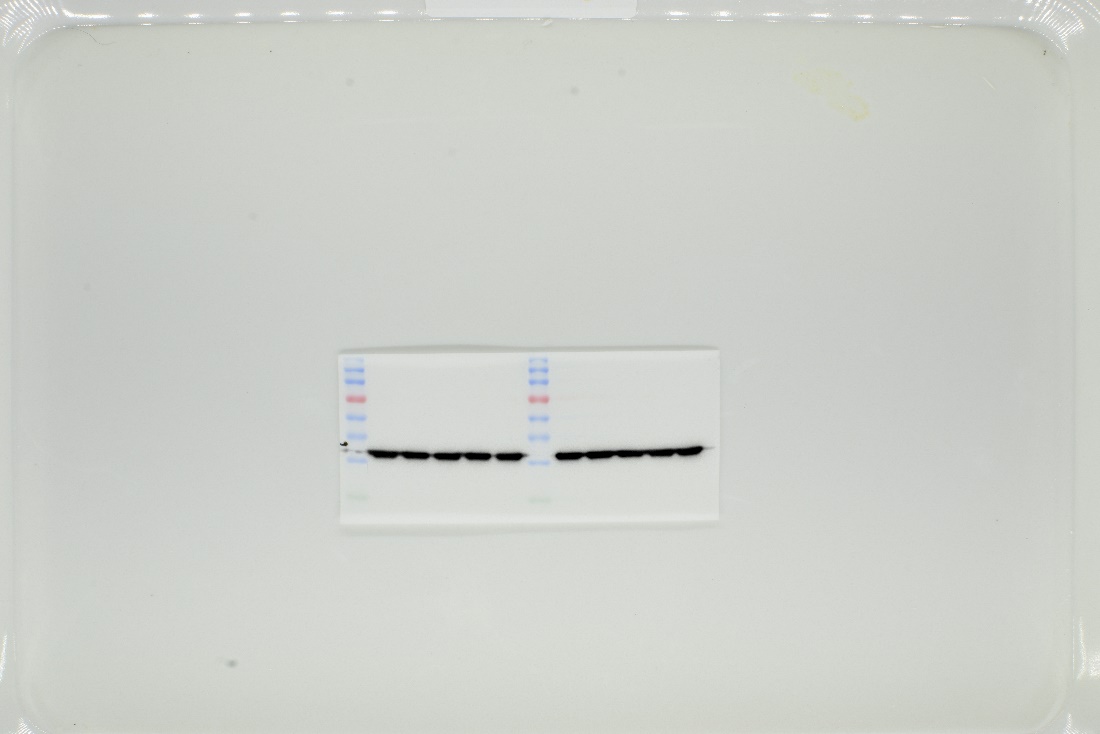


**Figure S7.** Full scan of the entire original gel (GAPDH: Group 1 and 2).


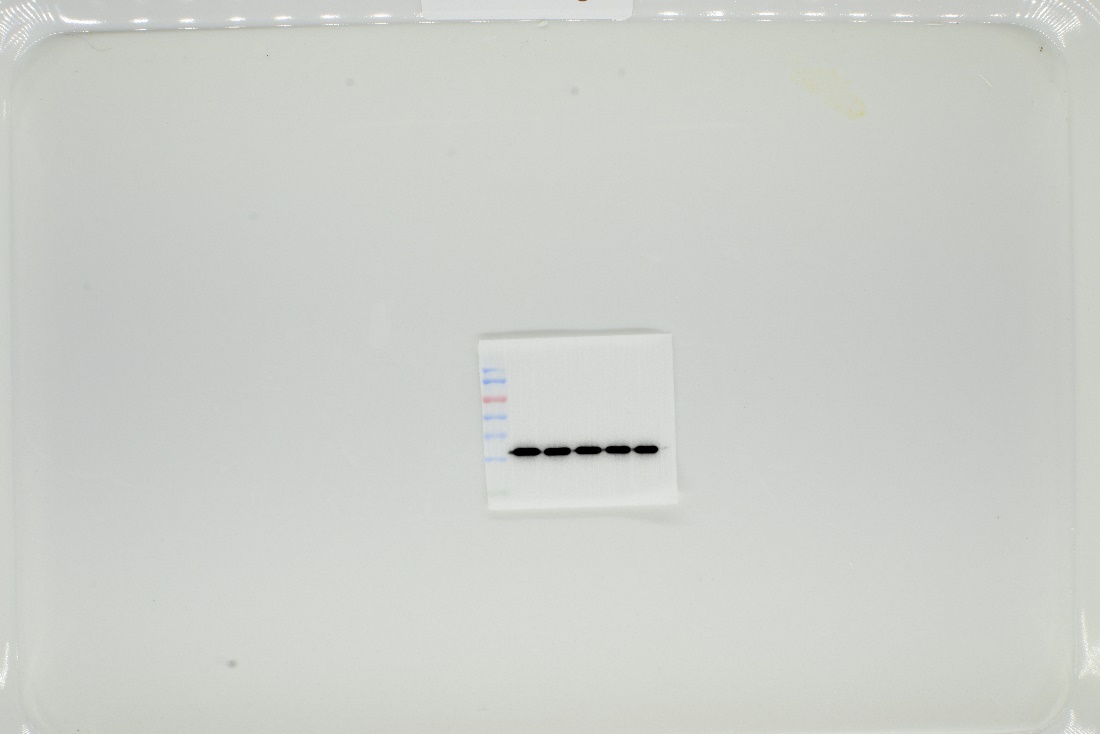


**Figure S8.** Full scan of the entire original gel (GAPDH: Group 3).
